# In Situ Scanning Transmission Electron Microscopy Calcination of Palladium Nitrate Supported on Zinc Oxide

**DOI:** 10.1002/smsc.202400048

**Published:** 2024-05-16

**Authors:** Ansgar Meise, Marc Heggen, Rafal E. Dunin‐Borkowski, Marc Armbrüster

**Affiliations:** ^1^ Ernst Ruska‐Centre for Microscopy and Spectroscopy with Electrons Forschungszentrum Jülich GmbH 52425 Jülich Germany; ^2^ Faculty of Natural Sciences, Institute of Chemistry, Materials for Innovative Energy Concepts Chemnitz University of Technology Straße der Nationen 62 09107 Chemnitz Germany

**Keywords:** calcination, environmental scanning transmission electron microscopy, in situ, palladium nitrate

## Abstract

Metallic nanoparticles are essential materials in various applications, such as nanomedicine, nanotechnology, and catalysis. While it is known that their catalytic performance is determined by their microstructure and thus by their preparation, the influence of an important but commonly used calcination treatment during nanoparticle preparation on their properties is often overlooked. Herein, structural and morphological changes during the calcination of Pd(NO_3_)_2_/ZnO are studied systematically by performing in situ heating experiments mimicking typical preparation conditions, by employing environmental scanning transmission electron microscopy. The effect of different calcination parameters on Pd(NO_3_)_2_/ZnO is explored and guidance to enhance control over the preparation of small supported nanoparticles is provided. It is shown that a calcination treatment of Pd(NO_3_)_2_/ZnO between 200 and 400 °C for 15–120 min is ideally suited to obtain particles smaller than 4 nm with a narrow size distribution.

## Graphical abstract

Based on a systematic investigation of calcination parameters, highly dispersed PdO particles smaller than 4 nm are studied during calcining at 200–400 °C for 2 h. Environmental scanning transmission electron microscopy reveals that temperatures above 400 °C promote particle mobility and agglomeration, leading to an increase in particle size.

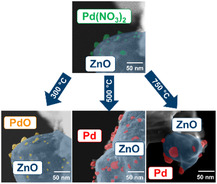

## 1 Introduction

Metallic nanoparticles supported on metal oxides are essential catalyst materials in many different reactions. Cobalt nanoparticles in the Fischer–Tropsch process^[^ ^1^, ^2^ ^]^ or iron nanoparticles in the Haber–Bosch process^[^ ^3^ ^]^ are prominent examples for supported metal nanoparticles employed in catalytic reactions. Their catalytic performance is largely determined by the material properties of the support‐particle system. As the particle morphology, particle distribution, and chemical composition are determined during synthesis,^[^ ^4^ ^]^ the controlled preparation of supported particles is often as important for catalytic performance as macroscopic reaction variables such as feed, temperature, and pressure.

Calcination treatments are central reaction steps in the synthesis of metallic particles.^[^ ^5^, ^6^, ^7^, ^8^ ^]^ During such a thermal treatment, impurities and volatile substances such as carbonates and nitrates are removed by heating the particles in an oxygen‐containing atmosphere. This heat treatment may induce changes in the composition, morphology, size, distribution, and crystal structure of the nanoparticles. The effect of calcination on metallic nanoparticles has been the subject of numerous studies in the past.^[^ ^9^, ^10^, ^11^, ^12^, ^13^, ^14^, ^15^, ^16^, ^17^ ^]^ As the anticipated particle changes take place on the scale of nanometers, characterization can be a methodological challenge. A frequently used characterization method is transmission electron microscopy (TEM) due to its high spatial resolution and different spectroscopic capabilities. In most TEM studies, the nanoparticles have been characterized before and after calcination. Although such ex situ studies are well suited for investigating postreaction states, dynamical changes and intermediate states formed during calcination are not resolved. Environmental scanning transmission electron microscopy (E‐STEM) has excellent prerequisites to study dynamical changes during the calcination treatment of nanoparticle catalysts, as it combines the spatial resolution required to image nm‐sized particles with gas atmospheres at pressures up to 2000 Pa.^[^ ^18^ ^]^ The residual pressure difference to ambient reaction conditions and its effect on reactions has been studied for many years. In the 1920s, Langmuir^[^ ^19^ ^]^ and Hinshelwood^[^ ^20^ ^]^ established a quantitative theory of catalysis, which highlights the significance of the partial pressure and thus the chemical potential in catalysis. According to their work and the standard model,^[^ ^21^ ^]^ most important variables for catalysis kinetics, such as coverage and reaction speed are determined quantitatively by the chemical potential. Although it was not considered by the Langmuir–Hinshelwood model, there is a dynamic interaction between the catalyst and the local chemical potential of the reactants, which triggers material changes.^[^ ^21^ ^]^ The so‐called “pressure gap” describes the pressure‐related discrepancy of reaction kinetics in heterogeneous catalysis. This phenomenon leads to different kinetics over different pressure ranges and also applies during synthesis. In the present study, E‐STEM is employed to study dynamic behavior during the calcination of metallic nanoparticles. The pressure gap is addressed by comparison to the in situ characteristics of the materials during thermal analysis at normal pressure.

### 1.1 Ex and In Situ Calcination Experiments

To date, there have been only few attempts to perform calcination experiments on metallic nanoparticles using in situ TEM.^[^ ^22^, ^23^, ^24^, ^25^, ^26^, ^27^, ^28^, ^29^ ^]^ Epicier et al.^[^ ^23^ ^]^ studied the calcination and reduction of Pd nanoparticles on *δ*‐alumina between 150 and 450 °C using E‐STEM. Their work revealed that particle mobility is limited at calcination temperatures below 450 °C and the observed size increase is mainly due to Ostwald ripening. Furthermore, they show that E‐STEM is a suitable method to study the in situ evolution of, for instance, nanoparticle size during the calcination of nanoparticles, with good correspondence to conventional ex situ TEM studies. An in situ TEM study by Zhang et al.^[^ ^29^ ^]^ reported the structural evolution of supported palladium–ceria core–shell nanoparticles at calcination temperatures between 500 and 800 °C. At 500 °C, they observed the separation of individual Ce atoms from smaller nanoparticles to form noncrystalline “clouds.” At 650 °C, these clouds restructured to clusters, which anchored to surrounding larger nanoparticles. Fiordaliso et al.^[^ ^28^ ^]^ investigated the morphological changes of GaPd_2_ supported on SiO_2_ during calcination at 260 °C and reduction at 550 °C using ETEM. These ETEM results revealed that the size and distribution of intermetallic nanoparticles that are employed in CO_2_ hydrogenation were determined upon calcination. No significant change was observed during reduction or hydrogenation. However, all of these articles report features that can only be revealed in situ, emphasizing the need for in situ TEM investigations for a complete understanding of dynamical changes of supported nanoparticles during calcination.

A highly interesting material system prepared by a preceding calcination step comprises intermetallic ZnPd nanoparticles supported on ZnO. This material system exhibits excellent catalytic properties in several heterogenous catalysis reactions, such as methanol steam reforming,^[^ ^30^ ^]^ the reverse water–gas‐shift reaction,^[^ ^31^ ^]^ and selective hydrogenation reactions.^[^ ^32^, ^33^, ^34^ ^]^ Classically, Pd/ZnO is used as a precursor for the formation of ZnPd/ZnO by reactive metal–support interaction^[^ ^35^ ^]^ and is prepared by incipient wetness impregnation (IWI) or wet impregnation (WI) using Pd(NO_3_)_2_^[^ ^36^ ^]^ (by far the most often used route), Pd acetate,^[^ ^37^ ^]^ Pd acetylacetonate,^[^ ^38^ ^]^ Pd(NH_3_)_4_(NO_3_)_2_,^[^ ^39^ ^]^ PdCl_2_,^[^ ^40^ ^]^ or Na_2_PdCl_4_^[^ ^41^ ^]^ as precursors. This step is then followed by drying and often subsequent calcination at temperatures between 400 and 500 °C for 3–16 h.^[^ ^32^, ^36^, ^42^ ^]^ Since the original synthesis description by Iwasa^[^ ^36^ ^]^ (IWI, aqueous Pd(NO_3_)_2_, drying at 383 K overnight and calcination at 773 K for 3 h), several modifications have been reported. Castillejos‐Lopez et al. investigated the influence of different ZnO morphologies on the catalytic properties of ZnPd/ZnO using WI with Pd(NO_3_)_2_ and direct reduction.^[^ ^43^ ^]^ The substitution of Pd nitrate by palladium acetate and water by chloroform (or acetone^[^ ^44^ ^]^) as a solvent is a further modification for the impregnation of ZnO.^[^ ^45^ ^]^ Here, also no calcination was involved, but the materials were directly reduced to form ZnPd/ZnO. Pfeiffer et al. used Pd acetate in toluene to impregnate ZnO, which was previously deposited on Al_2_O_3_ monoliths by washcoating.^[^ ^37^ ^]^ These were then calcined at 250 °C during which the Pd acetate decomposed into elemental Pd particles and traces of PdO.^[^ ^46^ ^]^ A higher calcination temperature of 500 °C was applied for 2 h after coimpregnation of Al_2_O_3_ monoliths by an aqueous solution of Pd(NO_3_)_2_ und Zn(NO_3_)_2_.^[^ ^47^ ^]^ The beneficial role of using PdCl_2_^[^ ^40^ ^]^ (with additional HCl) or Na_2_PdCl_4_^[^ ^41^ ^]^ as a precursor for WI due to the strong interaction with ZnO was reported. In all of these cases, it was not reported why the chosen calcination temperature and time were selected and how the material morphology changed during calcination. Although the initial synthesis route of Iwasa has proven useful to obtain materials with good catalytic activity,^[^ ^48^ ^]^ it is important to follow the process during synthesis and especially during calcination to improve the structure of the nanoparticle catalysts and their catalytic properties further. An influence of the calcination parameters on the microstructure of the supported particles is expected, but has so far not been investigated in detail (for simplicity, we use the term microstructure, even if the nanoscale is considered). The influence of the calcination procedure was investigated by Díez‐Ramírez et al. during the preparation of ZnPd/ZnO using two different precursors (Pd(NO_3_)_2_ or Pd(NH_3_)_4_(NO_3_)_2_) and a fast (going directly to maximum temperature) or slow (intermediate holding step at 180 °C) calcination procedure with a maximum temperature of 500 °C.^[^ ^39^ ^]^ The calcination temperature was chosen based upon thermogravimetric results, where the mass loss occurring at 127 and 227 °C assigned to loss of water and decomposition of the nitrate. X‐ray diffraction did not result in significant differences, but the CO_2_ hydrogenation properties were different, with a higher formation rate of methanol on the materials undergoing fast calcination. The differences were assigned to differences in metallic particles size, with the conclusion that the smaller particles resulting from slow calcination are less selective toward methanol but lead to CO formation.

As outlined above, many modifications to the initial synthesis route of Iwasa have been proposed. However, to date, only a few studies have investigated the preparation of supported Pd nanoparticles in a systematic way. In particular, the effects of calcination parameters on the microstructure have not been examined closely, let alone under in situ conditions. Although the work of Epicier et al.^[^ ^23^ ^]^ provided valuable information about the Pd/Al_2_O_3_ system during calcination, its significance for Pd particles on ZnO is limited. In addition, the work of Epicier et al. does not consider calcination temperatures above 450 °C, which are applied during some preparation protocols.^[^ ^36^, ^39^, ^42^ ^]^

The present systematic study aims to better understand the structural changes of palladium nitrate supported on zinc oxide in a calcinating environment and their effect on particle morphology and chemical properties. For this purpose, palladium nitrate is in situ heated from 100 to 500 °C at 1.5 Pa oxygen pressure using E‐STEM. The materials system is investigated simultaneously on the nanoscale, with transformation processes such as agglomeration and decomposition studied as a function of corresponding parameters during calcination. The E‐STEM capabilities that are used enable precise determination of material properties such as the decomposition temperature of palladium nitrate and zinc oxide. Ex situ experiments under identical calcination temperatures and dwell times but higher oxygen pressures are performed to investigate the influence of the pressure difference and the predictive power of the conducted E‐STEM experiments. Based on these results, this study aims to provide guidance for the enhancement of control over the preparation of PdO nanoparticles and their size.

## 2 Results and Discussion

### 2.1 Decomposition of Palladium Nitrate—Temperature Range from 100 to 200 °C

**Figure 1:**
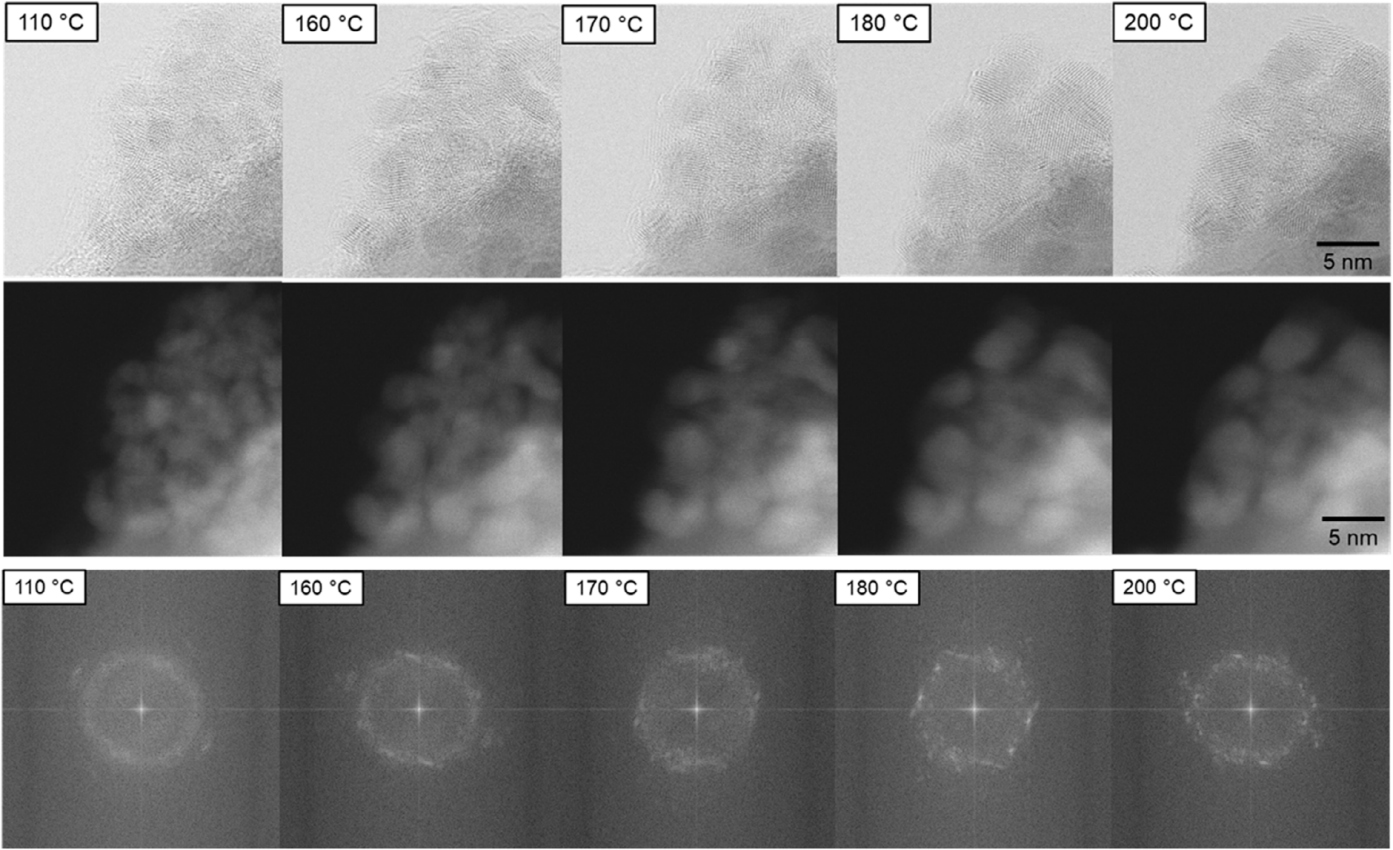
E‐STEM image series of the in situ calcination of Pd(NO_3_)_2_/ZnO in oxygen. Upper row: BF image series; middle row: corresponding DF image series; lower row: FFT series of corresponding particle cluster. New reflections appear at temperatures >170 °C.

**Figure** **1** shows an E‐STEM image series recorded during an in situ heating experiment from 110 to 210 °C in an oxygen atmosphere at 1.5 Pa. Further results are displayed in Figure S2, Supporting Information. This heating sequence was continued up to 500 °C at the same sample location illustrated, as shown in Figure 3 and S10, Supporting Information. Figure S18, Supporting Information, shows sample regions, which were not exposed to the electron beam during calcination. At 110 °C, the Pd(NO_3_)_2_ nanoparticles are anchored to the ZnO support.

**Figure 2:**
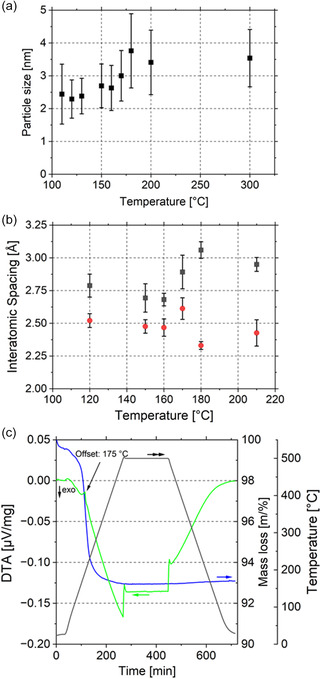
a) Particle size development as a function of temperature. The particle diameter is measured using two perpendicular lines: b) interatomic spacings along two directions (red and black). The measurements and directions are explained in Figure S4, Supporting Information; c) DTA experiment with indicated DTA (green), mass loss (blue), and temperature (black) graphs. The endothermic signal is indicated at 175 °C.

A comparison between dark field (DF) and secondary electron (SE) images in Figure S3, Supporting Information, indicates that the particles are almost exclusively found on the surface of the support because all of the particles that are visible in DF can be linked directly to particles located on the surface in the SE image. There, the palladium nitrate aggregated to form dense clusters of particles with strong adhesion to both the support and other particles. However, single particles can also be distinguished and no sintering effects are observed. The particle cluster is covered by an amorphous layer, which may be a contamination by residual hydrocarbons adsorbed during sample preparation. The layer can also originate from the nitrate ligands of palladium nitrate, which is a coordination polymer. The weak contrast of the layer in DF in Figure 1 indicates the absence of Pd. As the amorphous layer disintegrates as the temperatures increases, it may be incorporated into the particle. However, in order to ensure comparability of the particles, only structures that are clearly visible in the DF and crystalline in the bright field (BF) are considered. The palladium nitrate particle sizes were determined by measuring their diameters in the BF images using perpendicular lines (**Figure** **2**a). The diameters were averaged, with the error given by the standard deviation. The average size at 110 °C is $d_{110}=2.4\pm 0.9\text{ nm}$. The comparatively high standard deviation is due to the examination of <30 particles. The morphology of the particles and support material remains unchanged until ≈160 °C. At higher temperatures, first microstructural changes are initiated: Aggregating particles merge sluggishly with an increase in average particle size to 2.6±0.7 nm at 160 °C and 3.4±1 nm at 200 °C, as shown in Figure 2a. In the temperature range between 160 and 180 °C, strong particle growth is noticeable. Intriguingly, a further increase in temperature up to 300 °C is not accompanied by a further increase in particle size; the average particle size remains at 3.5±0.9 nm. The observed increase in particle size can be attributed to the calcination reaction converting palladium nitrate into palladium oxide. The temperature of the increase in particle size corresponds to the decomposition temperature of Pd(NO_3_)_2_ found in the differential thermal analysis (DTA) experiment displayed in Figure 2c. Here, the DTA results show the presence of an endothermic signal from the reaction at ≈175 °C and an endothermic shoulder from 165 to 200 °C giving similar temperature regimes to those in the in situ TEM experiment. Therefore, it can be concluded that the pressure gap is not significant in this case.

The thermal signal is also consistent with data obtained by Bruns et al.^[^ ^49^ ^]^ for anhydrous palladium nitrate. While their onset temperature of 165 °C exactly matches to the one found in this study, their maximal decomposition and final temperature are at higher temperatures. This thermal discrepancy can be attributed to the different morphology and heating rates. In Bruns’ work, the palladium nitrate crystals are in the μm range, whereas in the present in situ work the nanoparticles are in the nm range. Thus, the nanoparticles are prepared with a higher surface energy and are less stable, promoting their decomposition and explaining the lower temperatures. Bruns et al. heated the samples during DTA with a heating rate of 10 K min^−1^, which is larger than 2 K min^−1^ used in this study. As a result, the DTA signal could be delayed and only detected at higher temperatures.

A further indicator for the phase transformation from Pd(NO_3_)_2_ to PdO is the change in the interatomic spacings of the particles, which was monitored in two directions during heating as illustrated in Figure 2b. The directions and their labels are marked in Figure S4, Supporting Information. Here, a similar behavior is observed: at temperatures below 170 °C, the interatomic spacings remain unchanged at $g_{120\;{}^{\circ}C}=2.8\pm 0.1\;Å$ and $h_{120\;{}^{\circ}C}=2.5\pm 0.1\;Å$. Due to the complex crystal structure of palladium nitrate and an uncertain amount of water, a clear assignment of the interatomic spacings is difficult. However, the lattice spacings may correspond to anhydrous Pd(NO_3_)_2_ (1 1–3) lattice parameter of 2.39 Å, (1–2 0) of 2.47 Å, (1 0–3) of 2.56 Å, and (0 2 1) of 2.77 Å.^[^ ^49^ ^]^ For a hydrate crystalline, the Pd(NO_3_)_2_ · 2(H_2_O) (1 3 1) lattice spacings of 2.80 Å, (0 4 1) of 2.58 Å, (0 2 4) of 2.56 Å, (1 0 4) of 2.52 Å, (2 0 0) of 2.50 Å, (1 1 4) of 2.46 Å, and (0 4 2) of 2.41 Å are close to the measured lattice spacings.^[^ ^50^ ^]^ At temperatures above 170 °C, the spacings in direction g increase to $g_{210\;{}^{\circ}C}=3.0\pm 0.1\;Å$, while the average spacing in direction *h* shrinks to $h_{210\;{}^{\circ}C}=2.3\pm 0.1\;Å$. These spacings correspond to PdO (100) lattice plane spacing of 3.04 Å and (110) of 2.15 Å.^[^ ^51^ ^]^ The findings are supported by the corresponding fast‐Fourier transformation (FFT) images, which are shown for the selected particle cluster in Figure 1. During heating, the initial continuous, diffuse FFT ring, which indicates the presence of an amorphous phase, transforms to sharp reflections above the transformation temperature of 170 °C. This result supports the microstructural phase change from Pd(NO_3_)_2_ to PdO.

Thus, during the first heating stage an increase in particle size is observed, due to the decomposition of Pd(NO_3_)_2_ to PdO and simultaneous restructuring of the particles. A comparison between the TEM and DTA/thermogravimetry (TG) results reveals a negligible pressure gap.

### 2.2 Stable Calcination—Temperature Range from 200 to 400 °C

**Figure 3:**
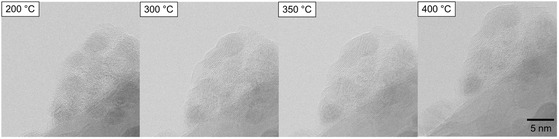
Series of BF E‐STEM images recorded over temperature range 200–400 °C. The morphology of the PdO/ZnO particles is largely unaffected by the temperature rise. A small increase in size of individual nanoparticles can be seen at a higher temperature.

Between 200 and 400 °C at a 1.5 Pa oxygen pressure, most PdO nanoparticles exhibit no significant growth in size. However, an increase in size can be observed in isolated cases. Quantification of the minor growth is challenging due to the tight aggregation of the nanoparticles and the lack of segmentation. As the particles are immobile, nanoparticle growth is most likely activated by surface diffusion and Ostwald ripening. Accordingly, in sample areas where the nanoparticles are further apart, as shown in Figure S5, Supporting Information, no significant growth can be seen. The brighter appearance of some nanoparticles at a higher temperature in Figure S5, Supporting Information, can be explained by more emitted SEs and a better signal‐to‐noise ratio. The ZnO support exhibits no decomposition or morphological changes. **Figure** **3** provides an overview of the morphology of the PdO/ZnO system over the temperature range between 200 and 400 °C.

The structure and morphology of the PdO/ZnO system is largely unaffected by the temperature rise. The presence of only small growth phenomena occurring at isolated nanoparticles indicates the stability of the system above 200 °C and supports the assumption that the discrete increase in particle size at 170 °C is associated with the phase transformation of Pd(NO_3_)_2_ into PdO.

These observations are in good correspondence with the work of Li et al.^[^ ^52^, ^53^ ^]^ For the conversion of glycerol to 1,2‐propanediol, they prepared a 5 wt% ZnPd/ZnO(‐3Al) catalyst via IWI using an acetone solution of Pd(C_2_H_3_O_2_)_2_. Subsequently, a two‐step calcination was performed, with the support first calcined at 350 °C for 5 h and the impregnated material calcined at 300 °C for 2 h and then reduced at 400 °C for 2 h. The as‐formed average particle size was between 1.4 nm for the ZnPd/ZnO(‐3Al) particles and 4.5 nm for ZnPd/ZnO. The sizes of the particle are consistent with the data obtained from the present in situ experiments, despite the use of a different precursor and atmosphere. The measured particle size in the in situ experiment at 300 °C is 3.5 ± 0.9 nm, as displayed in Figure 2a. Correspondence with the ex situ experiments of Li et al. suggests that neither dwell times below 120 min nor temperatures below 400 °C have a significant effect on particle size. It also indicates that there is a wide stability range for the calcination process, which generates uniform, small particles, making the preparation process robust and reproducible. Additionally, these experiments underline the small (if present at all) pressure gap of the present E‐STEM in situ calcination experiments. This is an interesting result because there is a tremendous difference in pressure between the E‐STEM experiments and laboratory calcination experiments: The in situ TEM O_2_ pressure regime is around $p_{\text{ESTEM}}=1.5\;\text{Pa}$, while the O_2_ partial pressure in ambient air is about $p_{\text{air}}=20000\;\text{Pa}$. Despite the large difference between the O_2_ pressure levels, and thus the lower chemical potential of oxygen, the PdO particles show similar behavior and do not agglomerate. Based on these results, it can be concluded that the O_2_ partial pressure has a minor impact on the mobility of the particles.

### 2.3 Agglomeration and Transformation of Nanoparticles—Temperature Range from 400 to 500 °C

**Figure 4:**
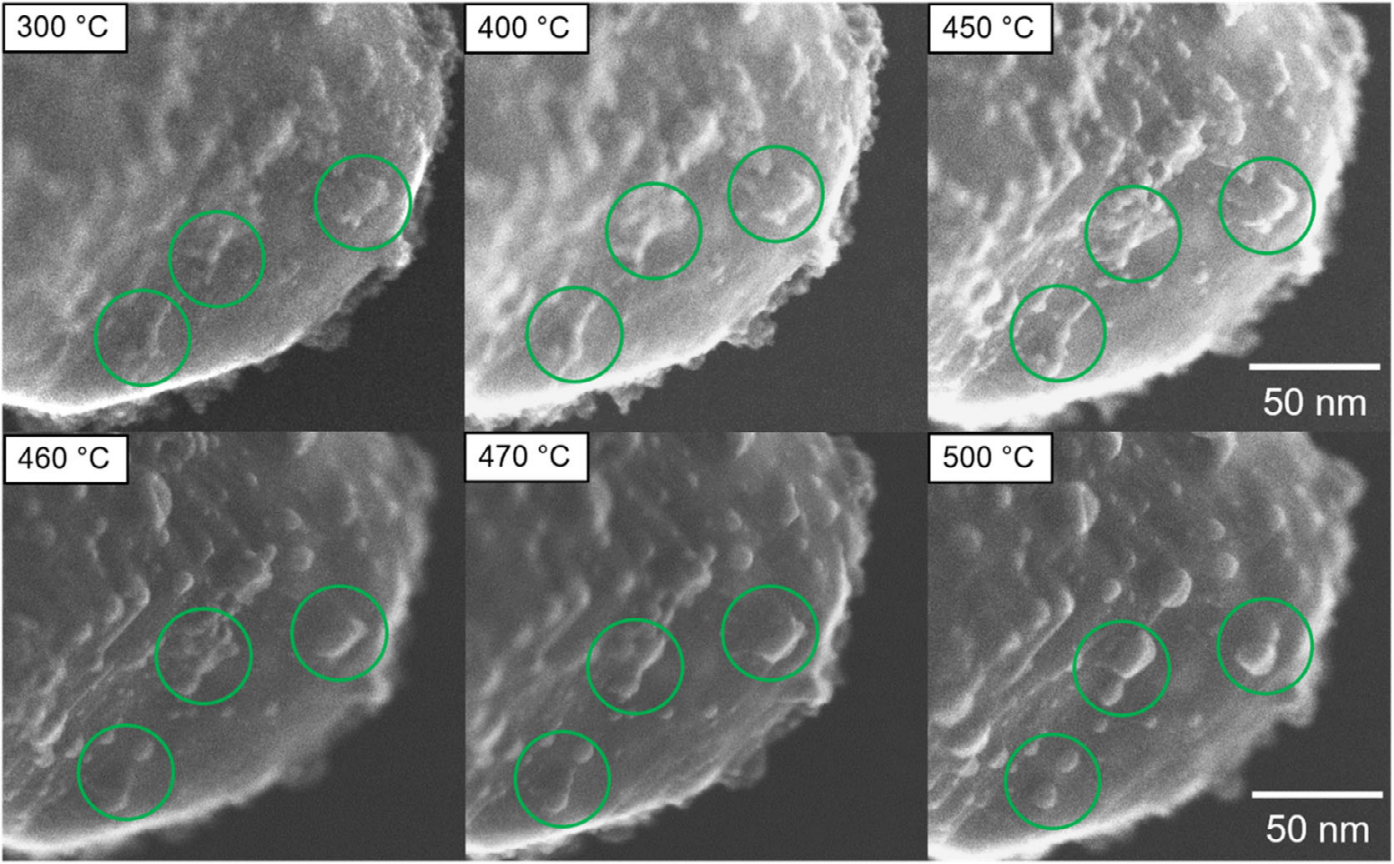
SE E‐STEM image series recording during heating, showing structural changes triggered by the temperature rise. Regions of interest are highlighted by green circles.

**Figure** **4** shows E‐STEM results obtained from an in situ calcination experiment performed between 300 and 500 °C at 1.5 Pa oxygen pressure. Figure S19, Supporting Information, shows sample regions, which were not exposed to the electron beam during calcination. Three regions of interest, which are marked by green circles, show the evolution of PdO nanoparticles during heating. Over this temperature range, microstructural changes of the PdO/ZnO system are triggered. Careful observation of the surface during heating first reveals particle movement, rounding, and agglomeration at temperatures between 450 and 460 °C.

**Figure 5:**
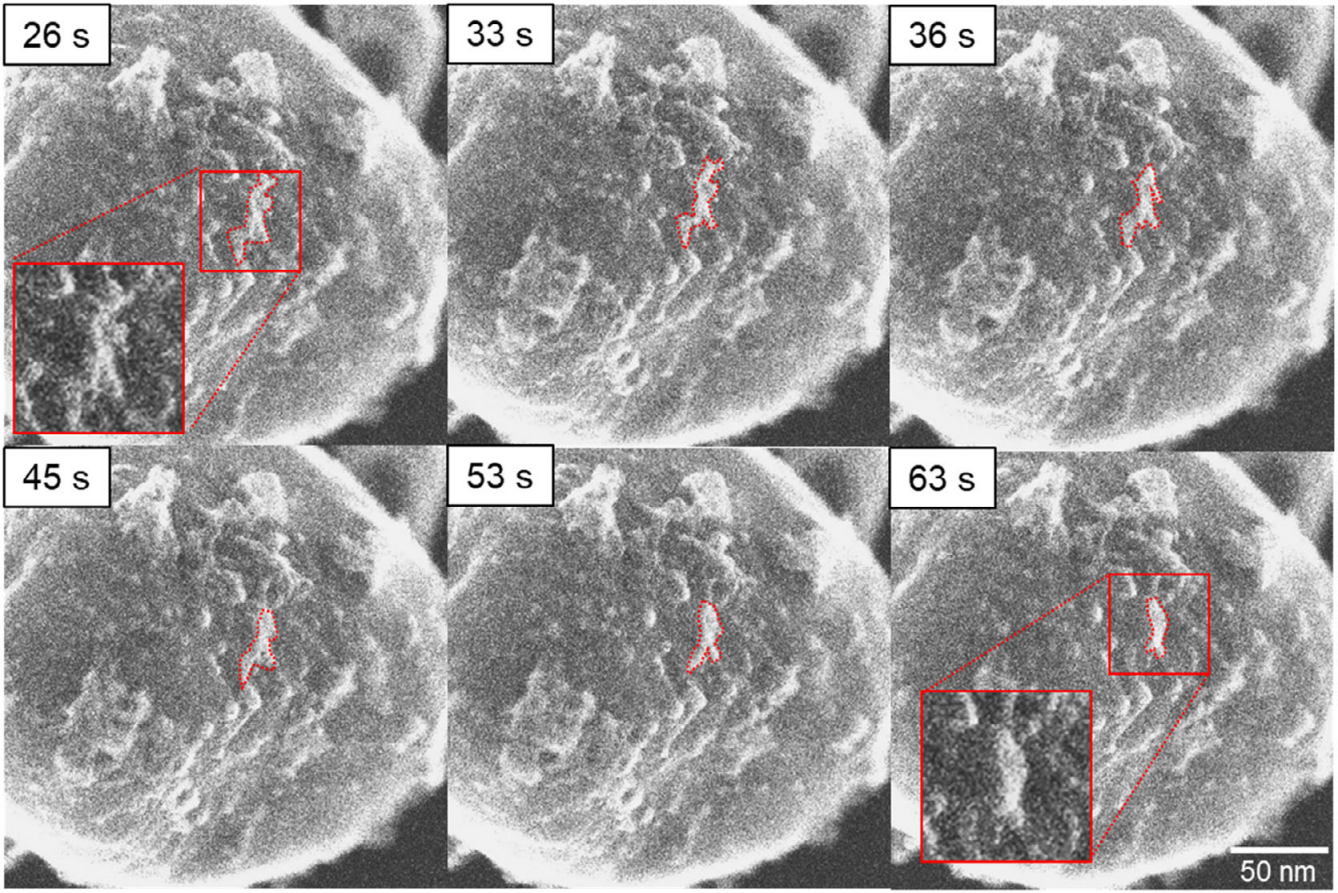
SE E‐STEM image series of particle merging at 460 °C in oxygen. The time stamps indicate the period after 460 °C was reached. The red dotted lines mark a group of selected particles and highlight the movement and agglomeration of these particles over time. The insets magnify regions of interest.

At 450 °C, the PdO particles shown in Figure 4 have a rough and uneven surface, suggesting the presence of particles in the marked regions. **Figure** **5** illustrates the evolution of a representative particle at 460 °C. After 33 s at 460 °C, surrounding particles begin to move across the surface toward the highlighted particle. Over the next 30 s, the particles agglomerate to form a larger particle, which becomes rounder due to a relative reduction in the surface (at 63 s). Agglomeration causes the nanoparticle to change its shape from an irregular to a compact and round shape. Intriguingly, the change in the particles’ morphology is nearly complete after the first 90 s at 460 °C. After this quick initial change, the particle morphology remains stable.

Each further temperature increase results in the abovementioned particle changes, leading to an iterative, discontinuous evolution of the particle–support morphology. At 500 °C, most of the particles have spherical shapes and have increased in size, lowering the specific surface area. At some locations, isolated and thus smaller particles exist. Thirty‐five particles smaller than 5 nm were measured to have an approximate distance to their closest neighbors of $d_{\text{neigh}}=5.6\pm 2.4\;\text{nm}$. This is distinctly larger than the average approximate particle distance at 400 °C. Qualitatively, this distance depends on the temperature and size of the particle, as well as on the sizes of the surrounding particles and the support morphology. Quantification of this distance will be subject of future investigations.

Ex situ calcination experiments at ambient air pressure were performed at 500 °C with a 180 min dwell time. Different PdO loadings (1%, 3%, and 5%) on ZnO were calcined under identical conditions. Representative SE images of samples with different loadings and their particle size distributions are shown in Figure S6, Supporting Information. Particle size histograms for the corresponding loadings are shown in Figure S7, Supporting Information. The average particle size at 500 °C and 3% loading is $d_{3\%,500}=8.3\pm 4.2\text{ nm}$. It is remarkable that the 1% and 5% loadings correspond to similar average particle sizes at 500 °C, with $d_{1\%,500}=8.3\pm 4.4\;\text{nm}$ and $d_{5\%,500}=7.6\pm 2.5\;\text{nm}$. The particles that were calcined at 500 °C are substantially larger than those prepared by Li et al. which were calcined and reduced at temperatures below 400 °C and exhibited an average particle size of below 4.5 nm.^[^ ^52^, ^53^ ^]^

Several conclusions can be drawn from these observations. First, differences in Pd loading of between 1 and 5 wt% have only minor effects on the average particle size.

A calcination temperature above 400 °C is likely to be the driving force for particle growth. The surface mobility of nanoparticles at temperatures above 460 °C observed in the in situ TEM calcination experiments indicates agglomeration as a growth mechanism, as shown in Figure 5. For closely spaced and aggregating nanoparticles, where surface diffusion is energetically favorable, Ostwald ripening cannot be excluded as mechanism for particle growth. Ostwald ripening as a mechanism for particle growth was also found in the work of Epicier et al. for the calcination of palladium nitrate on δ‐alumina at temperatures of up to 450 °C. As 400 °C is the onset temperature for decomposition of PdO nanoparticles in oxygen atmosphere,^[^ ^54^, ^55^ ^]^ the underlying reason for the change of morphology is the start of the mobility of atoms in PdO. The good correspondence between ex and in situ experiments underlines the small “pressure gap”. These results indicate that, when aiming at small particles, calcination temperatures should not exceed 400 °C. Under these conditions, the resulting small and well‐dispersed nanoparticles are promising to have a high active surface area, good catalytic performance, and high mass activity.

### 2.4 Formation of ZnO Rods—Temperature Range from 400 to 500 °C

**Figure 6:**
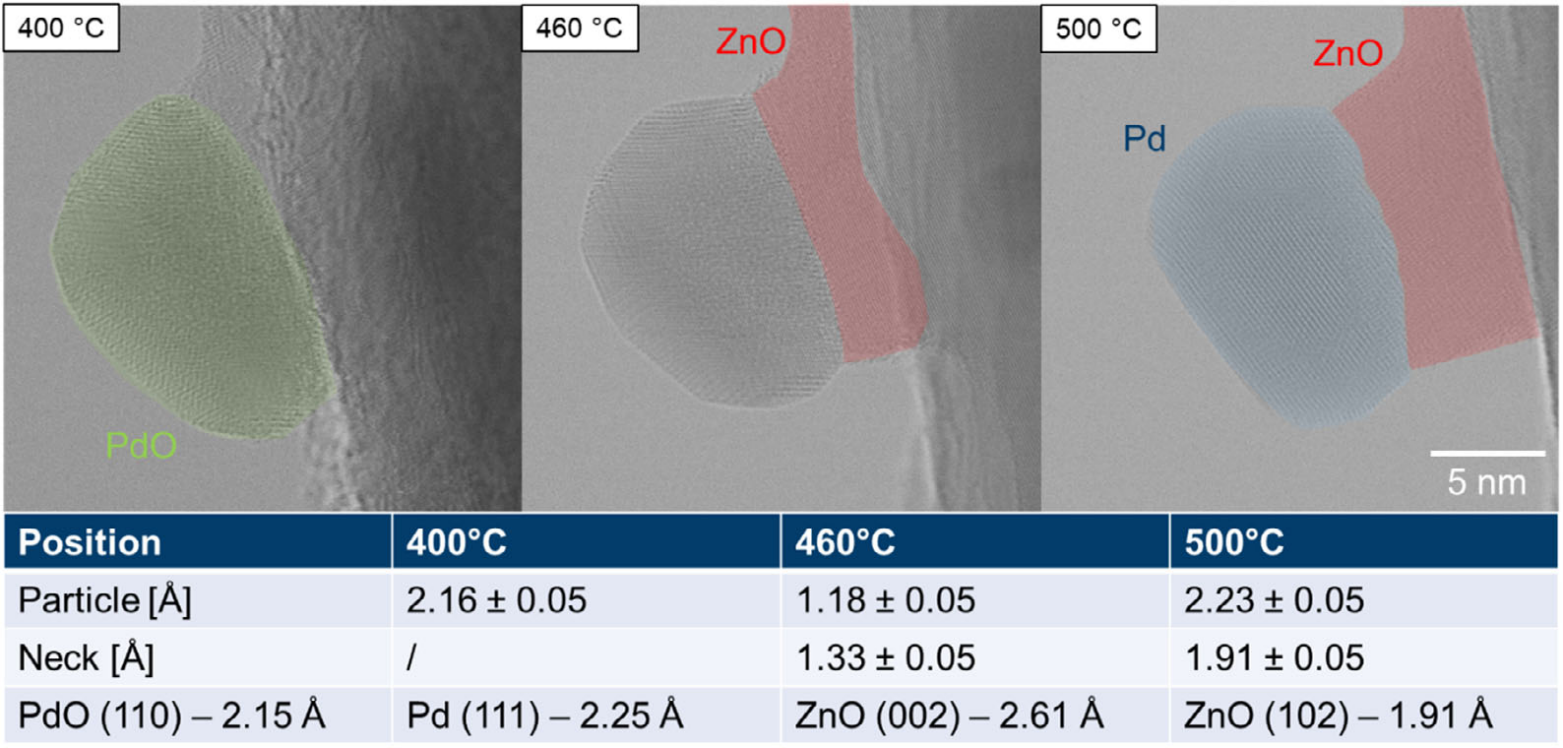
Phase identification via determination of lattice spacings. Corresponding FFT images are shown in Figure S16, Supporting Information; PdO (110) highlighted in green, ZnO (002) and (102) in red, and Pd (111) in blue. The lattice parameters are taken from refs. [51, 56].

The calcination process was then studied at high magnification to show the structural evolution on the atomic scale between 400 and 500 °C at 1.5 Pa oxygen pressure. Figure S8, S9 and S10, Supporting Information, illustrate the morphological evolution of the particles. The PdO particle shown in **Figure** **6** is polycrystalline and anchored to a flat ZnO support edge at 400 °C. During heating, the PdO restructures, forms surface facets at 460 °C, and develops a single‐crystalline structure, as underlined by a change in FFTs, which are shown in Figure S16, Supporting Information. At 400 °C, the lattice spacing of the particle is $d_{\text{particle},400}=2.16\pm 0.05\;Å$, which corresponds to PdO (110) with $d_{\text{PdO}}=2.15\;Å$ ^[^ ^51^ ^]^ highlighted in green in Figure 6. At 460 °C the measured lattice spacing decreases to $d_{\text{particle},460}=1.18\pm 0.05\;Å$, most likely indicating a mixed phase during transformation to metallic Pd.

At 500 °C, the lattice spacing of the particle is $d_{\text{particle},500}=2.23\pm 0.05\;Å$, which is in close agreement with Pd (111), which has a lattice spacing of $d_{\text{Pd}}=2.25\;Å$,^[^ ^56^ ^]^ suggesting the decomposition of PdO to cubic Pd.

By taking these micro‐ and macroscale effects together, it can be shown that the PdO decomposition is accompanied by higher particle mobility and agglomeration which leads to an increase in particle size. Following decomposition of PdO particles, a neck forms between the particles and the support. The lattice spacings in the neck at 460 and 500 °C match the interatomic spacings of ZnO (002) and ZnO (102), and are thus identified as the hexagonal wurtzite form of ZnO, which is a typical structure for ZnO in nanorods.^[^ ^57^, ^58^ ^]^ This finding can be attributed to the known catalytic formation of ZnO nanorods^[^ ^59^, ^60^, ^61^, ^62^, ^63^ ^]^ and is likely to be triggered by the O_2_ atmosphere and elevated temperature. The observed formation process of ZnO nanorods under the present experimental conditions can be explained as follows: Due to the low partial pressure of O_2_, the ZnO may be partially reduced to oxygen‐deficient Zn_*x*_O (*x* > 1). As a consequence of the formation of lattice vacancies, Zn^*δ*+^ becomes mobile. Simultaneously, Pd activates oxygen from the atmosphere and acts as an oxygen donor, which oxidizes the neighboring Zn^*δ*+^ again. The conditions trigger the formation of an epitaxially growing nanorod, where length is determined by the temperature‐dependent equilibrium. This proposed mechanism is supported by the fact that nanorods are only found in the presence of Pd nanoparticles (Figure S11, Supporting Information). Taking the macro‐ and microscale results into account, the in situ TEM calcination experiments provide insight into microstructural changes during heating between 400 and 500 °C. The activation temperature for particle merging can be determined to be between 450 and 460 °C.

Results from a decomposition experiment, which was performed to a temperature of 800 °C in vacuum, can be found in Figure S12 and S13, Supporting Information. Above 660 °C, decomposition of ZnO is initiated, resulting in faceting and disintegration due to the formation of elemental Zn and O_2_.

## 3 Conclusions

The calcination of Pd(NO_3_)_2_ supported on ZnO has been followed in situ using E‐STEM. At approximately 170 °C, Pd(NO_3_)_2_ starts to decompose into PdO, triggering a growth of particles. A stable calcination window of between 200 and 400 °C is determined, over which the particle size hardly changes. Above 460 °C, PdO starts to decompose into elemental Pd, accompanied by an increase in particle mobility on the ZnO surface, particle agglomeration, and the formation of ZnO nanorods. These phenomena could only be resolved by the use of in situ electron microscopy. All of our in situ E‐STEM findings are consistent with results obtained from ex situ calcination experiments performed under laboratory conditions at ambient pressure. Despite the pressure difference between our E‐STEM experiments and ex situ experiments under laboratory conditions, Pd(NO_3_)_2_ calcination is not found to be sensitive to the pressure conditions and the conducted in situ E‐STEM experiments are well suited to the study of Pd(NO_3_)_2_/ZnO calcination. Supported palladium nitrate is best calcined between 200 and 400 °C for 15–120 min to obtain particles smaller than 4 nm with a dense size distribution. In order to preserve the particle‐support structure, the system should not be heated to temperatures above 600 °C. The insight gained from this study is expected to be of assistance for enhancing the control and reproducibility of future ZnPd particle preparation and the consistency of heterogenous catalysis reactions.

Our study reports the first in situ high‐resolution imaging of the Pd(NO_3_)_2_/ZnO system during calcination and new light is shed on the potential of in situ E‐STEM experiments to understand the complex structural and morphological changes during the growth of intermetallic nanoparticle catalysts. These findings are important to understand the relationship between synthesis conditions, microstructure, and performance of metallic catalytic materials and may lead to a systematic improvement of the synthesis conditions and catalytic performance of supported metal nanoparticles.

## 4 Experimental Section

4.1

4.1.1

##### Sample Preparation

Samples were prepared by incipient wetness impregnation following typical protocols in literature (e.g., ref. [64]) using ZnO (Thermo Scientific, 99.99%) and Pd(NO_3_)_2_ · *X*H_2_O (Sigma–Aldrich) dissolved in the appropriate amount in water. As Pd(NO_3_)_2_ holds an undefined amount of water, the Pd content was determined by thermogravimetry (NETSCH STA 449F3) from the mass loss during reduction in 10% H_2_/He at 40 mL min^−1^. This resulted in a residue of 42.71 wt%, corresponding to Pd(NO_3_)_2_ · 1.04 H_2_O. After impregnation, samples were dried at 110 °C for 5 days.

##### Sample Characterization: DTA Analysis

DTA/TG (NETSCH STA 449F3) was conducted in 20% O_2_/He with 40 mL min^−1^ and a heating/cooling rate of 2 K min^−1^ to record temperatures of ongoing processes and mass loss during calcination of dried samples up to 500 °C. Samples of around 100 mg were placed in Al_2_O_3_ crucibles; an empty Al_2_O_3_ crucible was used as reference. Measurements were corrected by subtracting blind measurements conducted with the to‐be‐used crucibles under identical conditions before loading the samples.

##### Sample Characterization: ICP/OES Analysis

Elemental analysis of the samples was conducted in triplicate by inductively coupled plasma/optical emission spectroscopy (ICP/OES) against a matrix‐calibrated standard (Vista RL, Varian). A fraction of each sample was dissolved in aqua regia, diluted with distilled deionized water, and acidified with HCl before analysis.

##### Sample Characterization: TEM Analysis

TEM samples of supported Pd(NO_3_)_2_/ZnO (3 and 5 wt% loading) were prepared applying two different techniques. During dry preparation the palladium nitrate powder was scooped with a dropper tip and gently blown on a microelectromechanical systems (MEMS) heating chip. Subsequently, the correct position of the sample on the chip, i.e., the presence of catalyst powder on the SiN‐windows on the MEMS heating chips, was checked using an optical microscope (as illustrated in Figure S14, Supporting Information). The HTN‐1010H MEMS heating chips were supplied by Norcada Inc., Edmonton, Canada. If the catalyst powder was not centered on the electron transparent windows and the heating spiral, the process was repeated.

During wet preparation, the sample powder was poured in ultrapure water and suspended by shaking. Using an Eppendorfer pipette with a 10 μL tip, the suspension was picked up and transferred on the MEMS chip applying the drop‐cast method. The sample was dried under ambient lab conditions. Again, inspections using an optical microscope as during dry preparation were applied. Both casting methods produced similar sample distributions on the MEMS chip. The specimens were stored in an evacuated desiccator until used. Shortly before transition into the electron microscope, the specimen were treated under UV light at 10% air pressure for 5 min and plasma cleaned for 10 s in Ar atmosphere in a sample cleaner Hitachi ZONETEM II and a Fischione Instruments 1020 plasma cleaner (E.A. Fischione Instruments Inc., Export, USA).^[^ ^65^ ^]^

The in situ calcination experiment was conducted using E‐STEM on a Hitachi HF5000 operating at 200 kV and equipped with a spherical aberration probe corrector. The applied DF detector collection angle is 40–200 mrad, and the bright field detection collection angle is 0–3.5 mrad.

The dose per frame *D*_f_ applied was calculated by $D_{f}=\left(I_{b}/A\right)\cdot t_{f}$,^[^ ^66^ ^]^ where *I*_b_ is the beam current, *A* is the frame are on the sample, and *t*_f_ is the frame time. As the electron emitter used is a cold field emission gun (FEG), the beam current decays over time and the values given should be considered a guideline only. The beam current was measured shortly after flashing the FEG. During image acquisition of high resolution images (Figure 1, 3, 6, and S2, S4, S8, S9, S10, Supporting Information), the following parameters were applied: a dose per frame of ≈$3.7\times 10^{8}\;e^{-}\;{Å}^{-2}$, a dwell time per pixel of $19.1\mu s{\text{ pixel}}^{-1}$, and a beam current of 60.8 pA. For images with lower magnification (Figure 4, 5, and S3, S5, S6, S11, S12, S13, Supporting Information), a dose per frame $<3.0\times 10^{8}\;e^{-}\;{Å}^{-2}$ and a dwell time per pixel of $38.1\;\mu s{\text{ pixel}}^{-1}$ were used.

The specimens were heated following a specific heating profile which is shown in Figure S15, Supporting Information. First, the temperature was increased from room temperature to 100 °C and held for 15 min. Subsequently, the temperature was risen by increments of ⋅T=10 °C and kept for 3 min, until 210 °C were reached. Subsequently, the temperature was increased to 400 °C, holding at 300 and 350 °C for 3 min in between. In the temperature range between 400 and 500 °C, the temperature profile was controlled in the same manner as between 100 and 210 °C. Subsequently, the sample was heated at 500 °C for 30 min. A heating rate of $r_{\text{heating}}=1\;{}^{\circ}{\text{C s}}^{-1}$ was applied for every heating step. Ultrapure oxygen (99.99%) was inserted into the microscope column with a mass flow $\dot{m}=1\text{sccm}$ resulting in a column pressure $p_{\text{column}}=2.0\times 10^{-2}\text{ Pa}$ and a corresponding specimen pressure of $p_{\text{specimen}}=1.5\;\text{Pa}$. The TEM in situ calcination was repeated 3 times with coinciding outcome. To reduce beam exposure, the regions of interest were exposed to the electron beam only for image acquisition and for microscope focus alignment. During each heating step, two images were captured at different magnifications with a capture speed of 20 s image^−1^. Due to image and video acquisition in one experiment, the region of interest was continuously exposed to the electron beam. These results are displayed in Figure 4 and 5.

The decomposition of ZnO was studied under the following conditions. The sample was heated in vacuum continuously from 500 to 650 °C, dwelling for 3 min at 600 °C. Between 650 and 800 °C, the temperature was increased in 10 °C increments which were held for 3 min each. A heating rate $r_{\text{heating}}=1\;{}^{\circ}{\text{C s}}^{-1}$ was applied during every heating ramp. An illustration of the heating ramp is given in Figure S16, Supporting Information.^[^ ^67^, ^68^, ^69^, ^70^ ^]^

##### Sample Characterization: Data Analysis

STEM data were processed using MSpowerpoint (MOS2019) and the ImageJ add‐on FIJI (Version .1.8.0_172). Graphs were created with OriginPro 2023.

## Conflict of Interest

The authors declare no conflict of interest.

## Supporting information

Supplementary Material

## Data Availability

The data that support the findings of this study are available from the corresponding author upon reasonable request.
