# Supplementary material for: In Situ Scanning Transmission Electron Microscopy Calcination of Palladium Nitrate Supported on Zinc Oxide
Source: Small Sci. 2024 May 16;4(8):2400048. doi: 10.1002/smsc.202400048 (PMC11935018; doi:10.1002/smsc.202400048)
Supplement: Supplementary file 1 — Supplementary Material [file SMSC-4-2400048-s001.zip › smsc.202400048-sup-0001-suppdata-S1.pdf]

Supporting Information

In Situ Scanning Transmission Electron Microscopy Calcination of Palladium Nitrate Supported on Zinc Oxide

Ansgar Meise, Marc Heggen, Rafal E. Dunin-Borkowski, Marc Armbrüster*

Sample characterisation

Samples were prepared aiming at a Pd-loading of 1 wt%, 3 wt% and 5 wt% Pd/ZnO. Elemental analysis resulted in 1.0(1) wt% 3.4(2) wt% and 5.5(1) wt% of palladium, thus very close to the nominal values which are used in the following. Powder X-ray diffraction analysis of the slightly grey samples after calcination showed only ZnO reflections and was consistent with a very small crystallite size and/or poor crystallinity of the PdO (**Figure S1**). Thus, the obtained sample material is very similar to that prepared by the incipient wetness approach in literature.

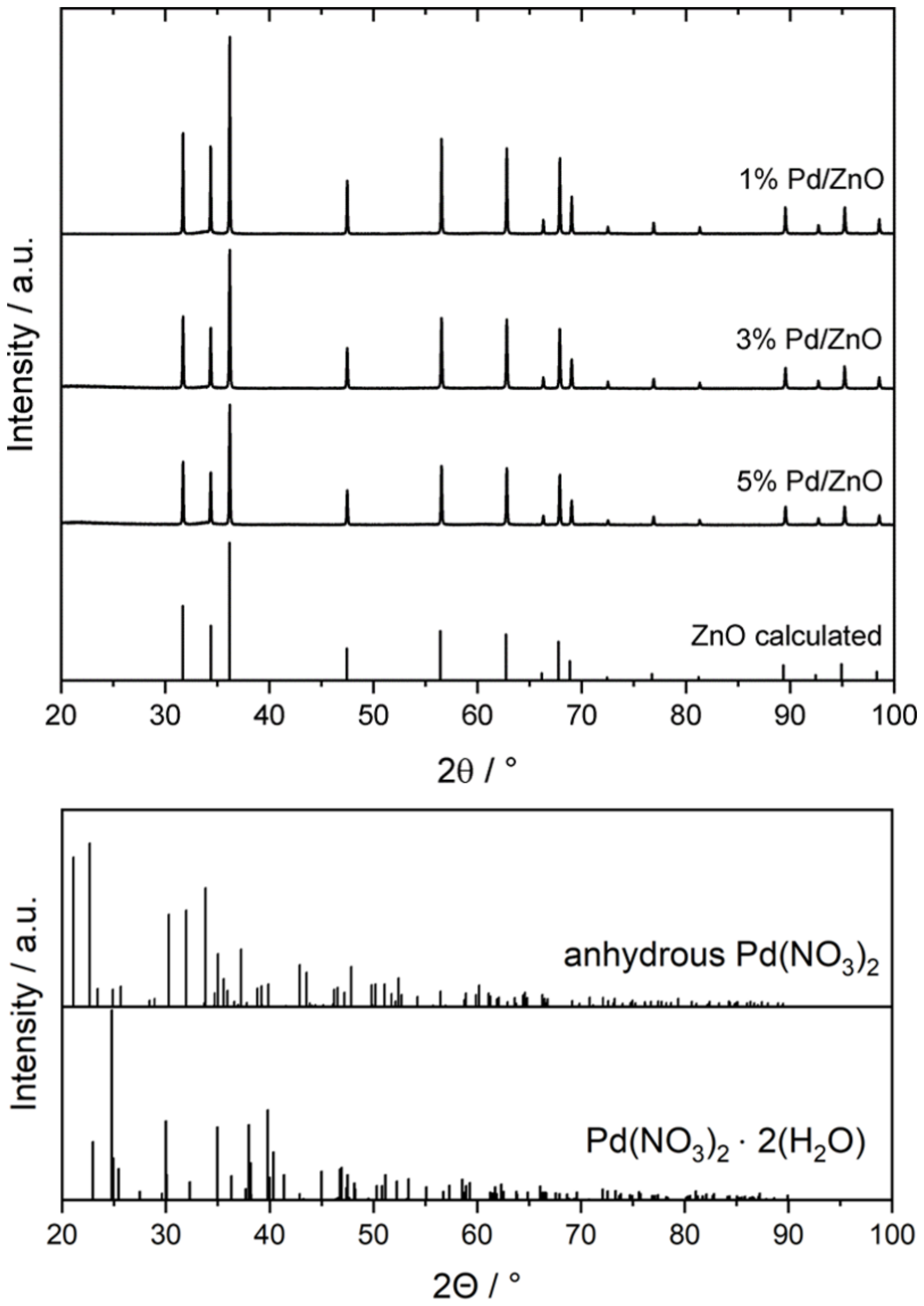

Figure S1: XRD results of different Pd-loading on ZnO (1%, 3% and 5%); calculated ZnO reflections are given for reference[67]; missing PdO reflections are due to small crystallite size and/or bad crystallinity. Diffraction patterns of anhydrous and water containing palladium nitrate are added for reference[49][50].

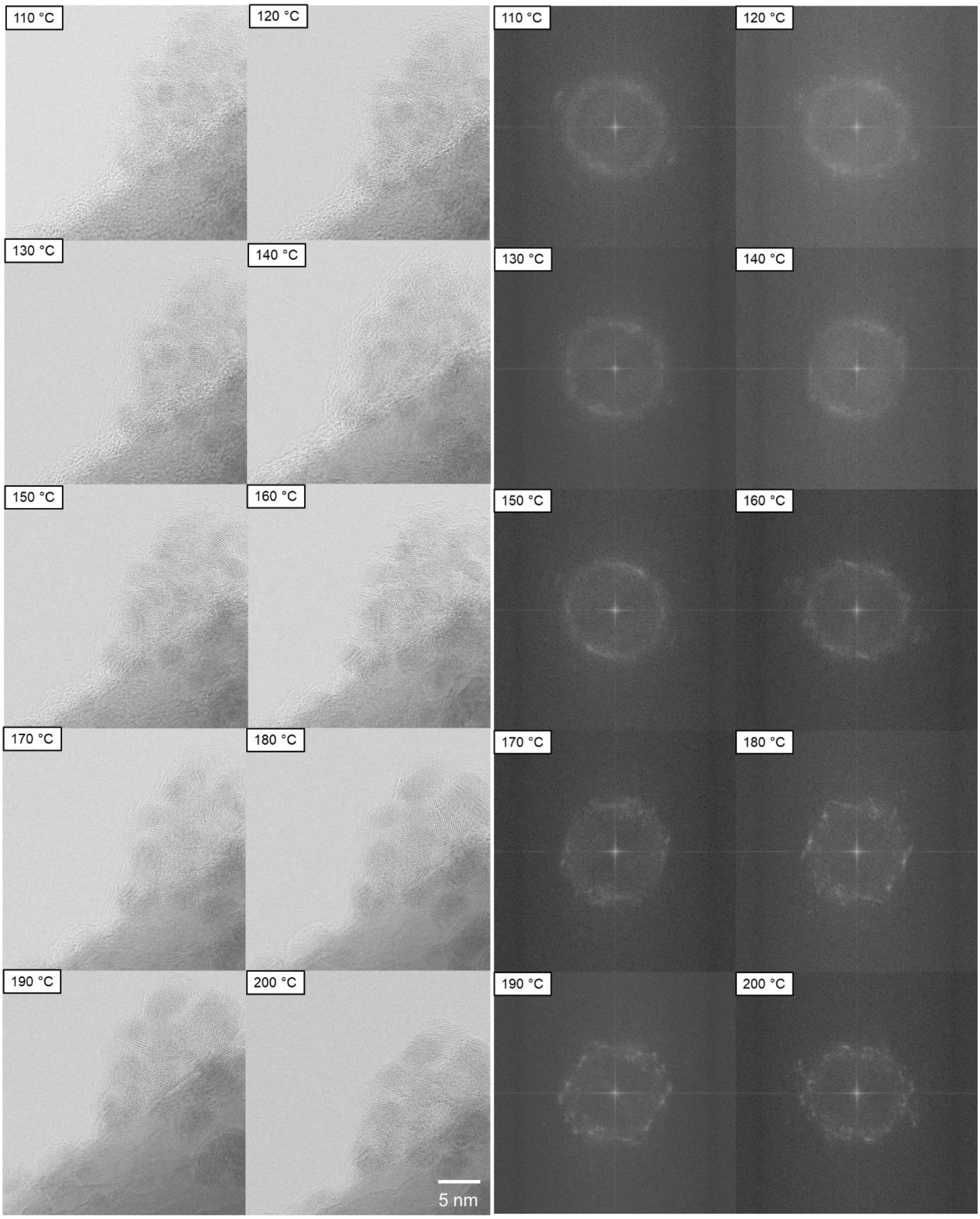

Figure S2: BF E-STEM image series and the corresponding FFTs of an *in situ* calcination experiment; the structural transformation of $Pd(NO_3)_2/ZnO$ to PdO/ZnO in oxygen is apparent.

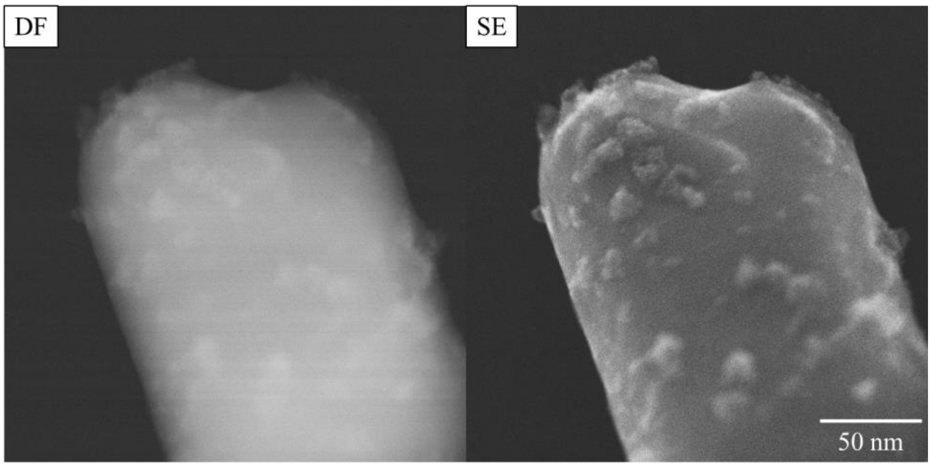

Figure S3: DF (left) and corresponding SE (right) image of a support-particle structure between $Pd(NO_3)_2$ and ZnO recorded at room temperature. A comparison between the locations of particles in the DF and SE images indicates that the majority of the particles is found on the surface.

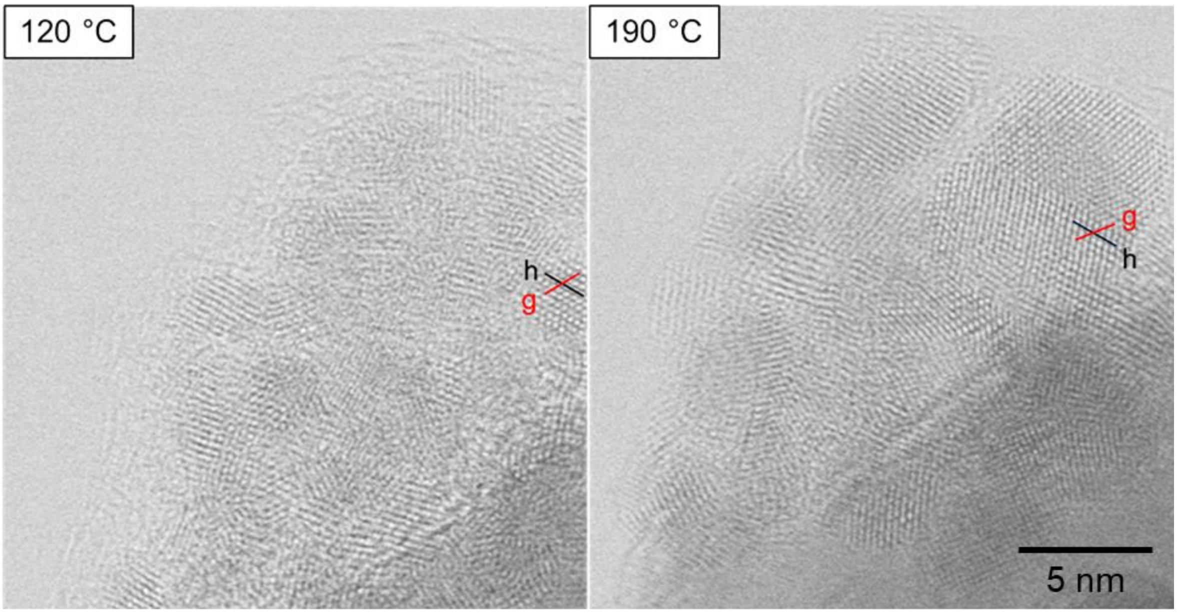

Figure S4: BF STEM measurements of interatomic spacings in the two marked directions labelled *h* and *g* on the same nanoparticle at 120 and 190 °C.

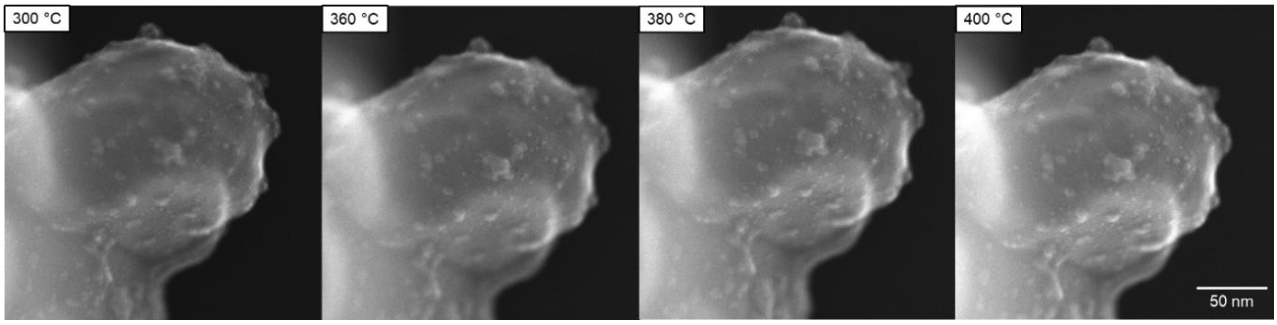

Figure S5: SE E-STEM image series of different heating steps between 300 and 400 °C. Neither particle movement nor agglomeration can be detected.

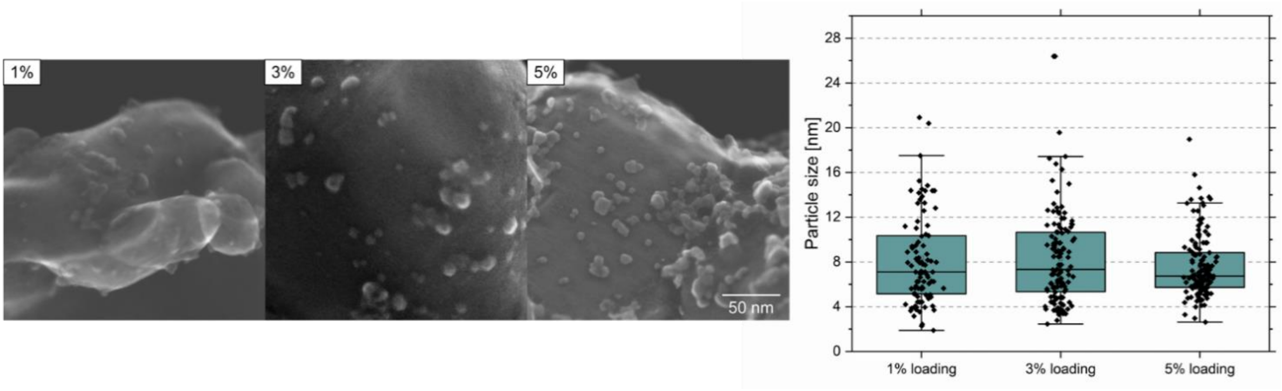

Figure S6: Left: Different PdO/ZnO loadings (left to right: 1%, 3% and 5%) of samples calcined at 500 °C for 180 min at ambient conditions. Right: Corresponding particle size distributions. The green boxes indicate the median of the upper and the lower half.

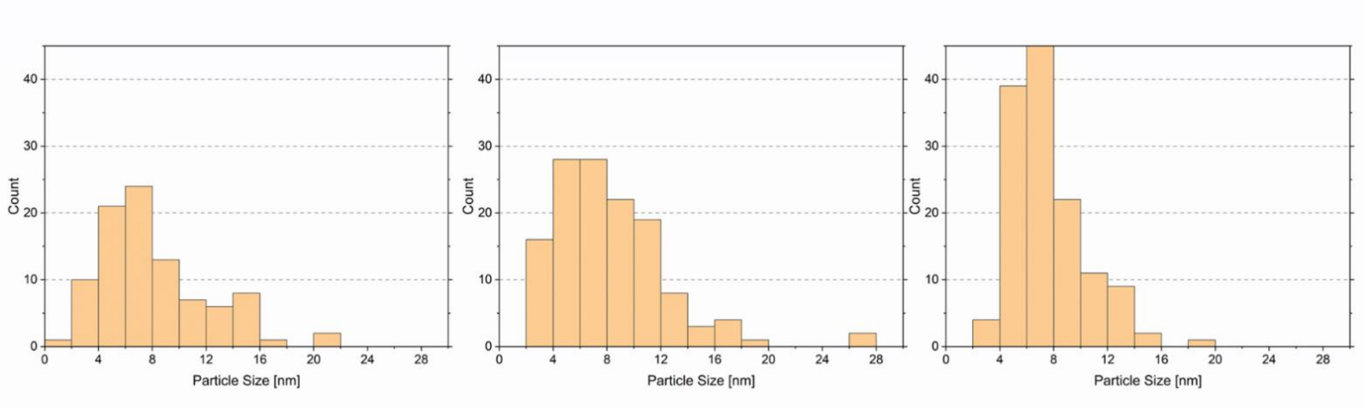

Figure S7: Particle size histograms of *ex situ* calcined PdO nanoparticles on ZnO with different loadings (left to right: 1%, 3%, 5%) performed under identical, ambient laboratory conditions (500 °C, 180 min dwell time, ambient air pressure).

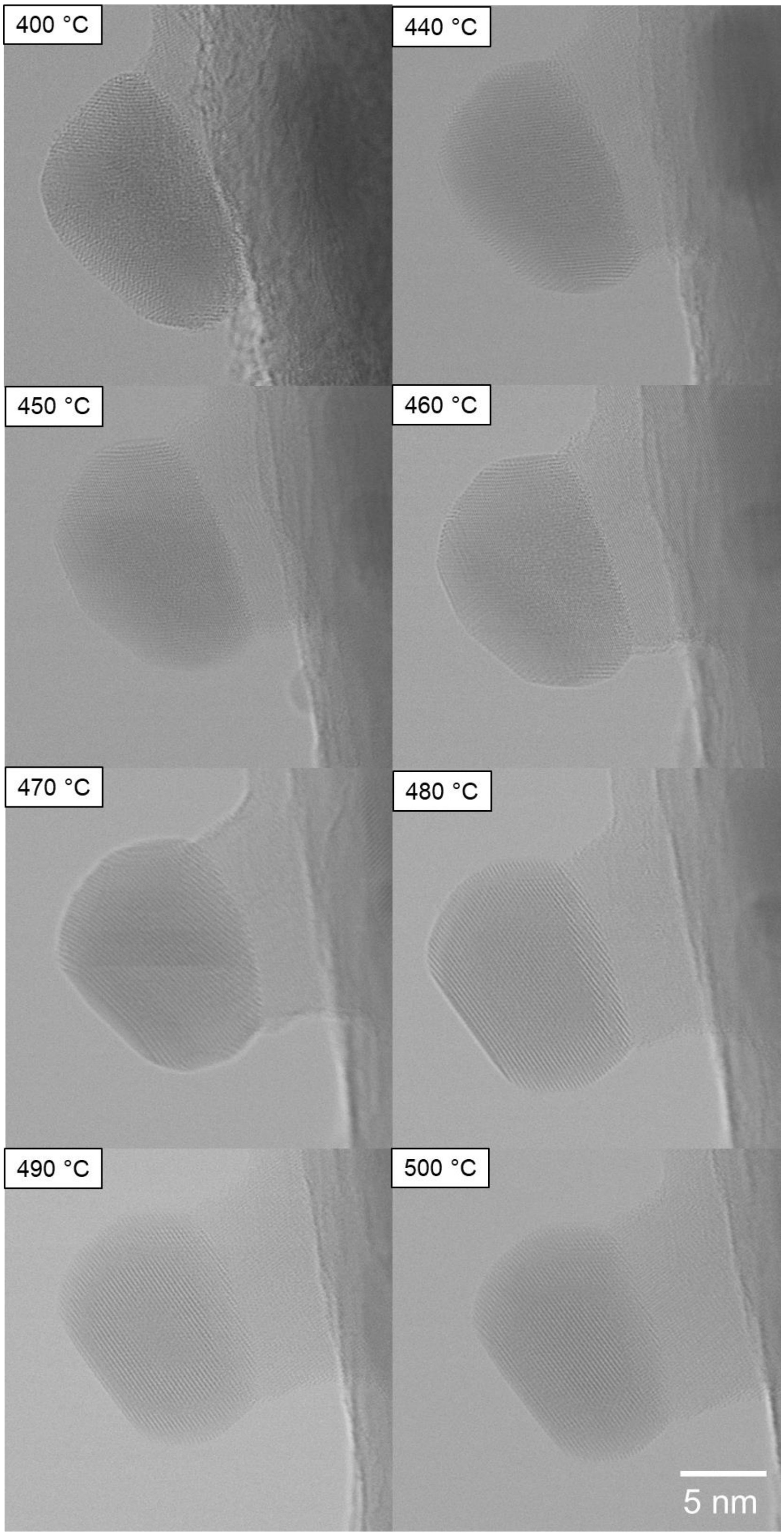

Figure S8: High-resolution BF E-STEM image series of the evolution of an individual particle during heating from 400 to 500 °C. The PdO particle and ZnO support form a mushroom-like structure.

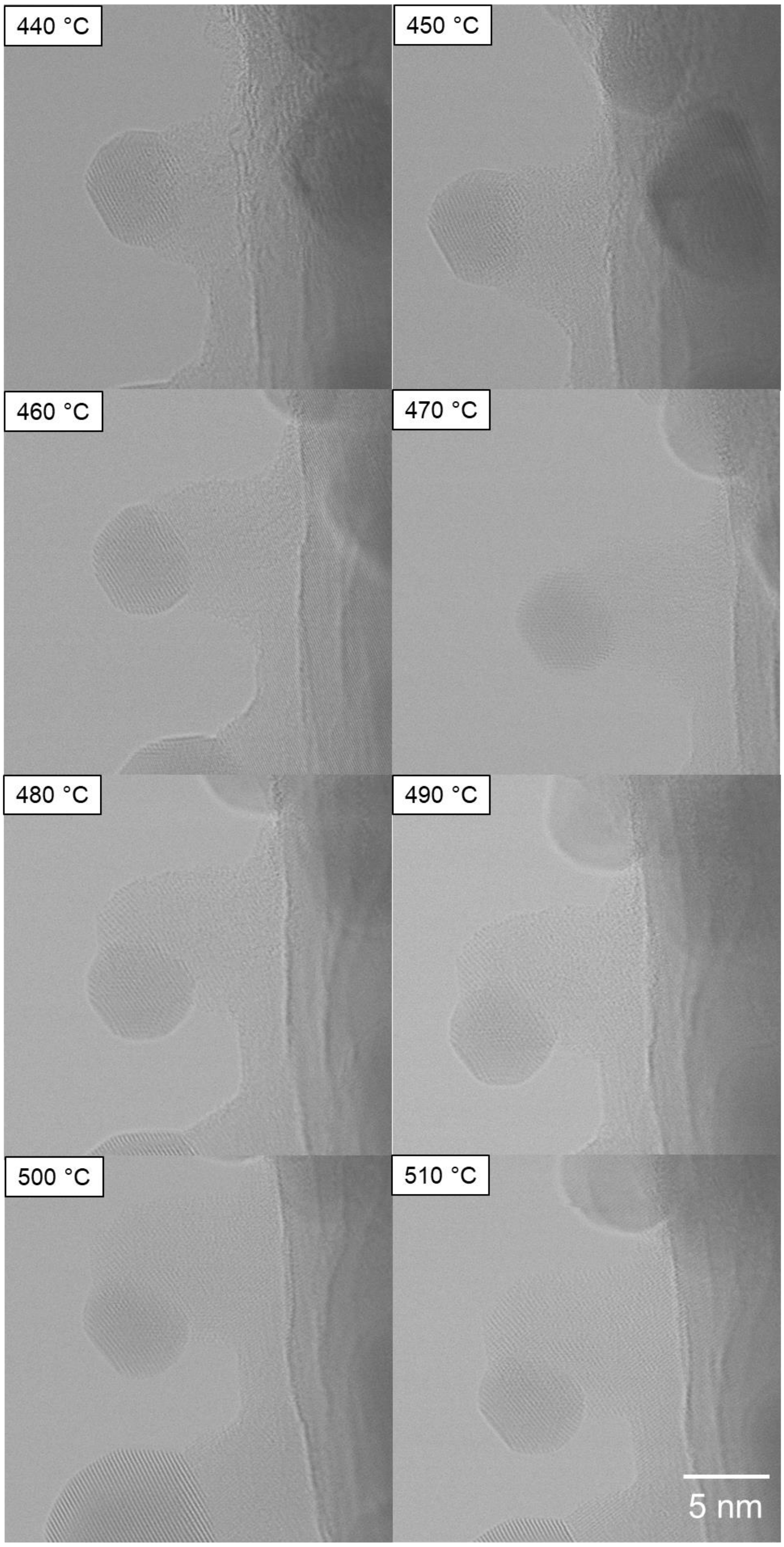
440 °C
450 °C
460 °C
470 °C
480 °C
490 °C
500 °C
510 °C
5 nm

Figure S9: High-resolution BF E-STEM image series of the evolution of an individual particle during heating from 400 to 510 °C. A mushroom-like structure and neck growth are observed.

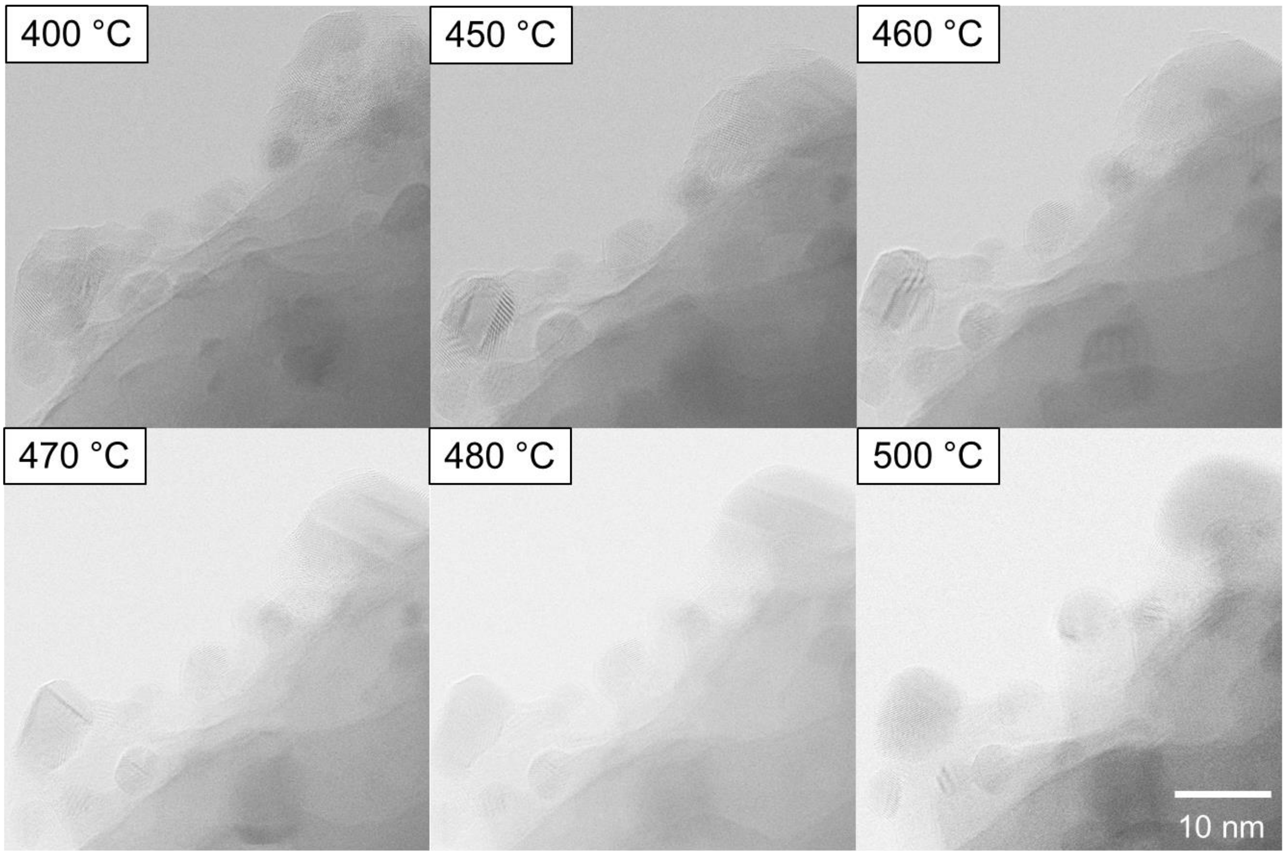

Figure S10: BF E-STEM images series of particle evolution while heating from 400 to 500 °C.

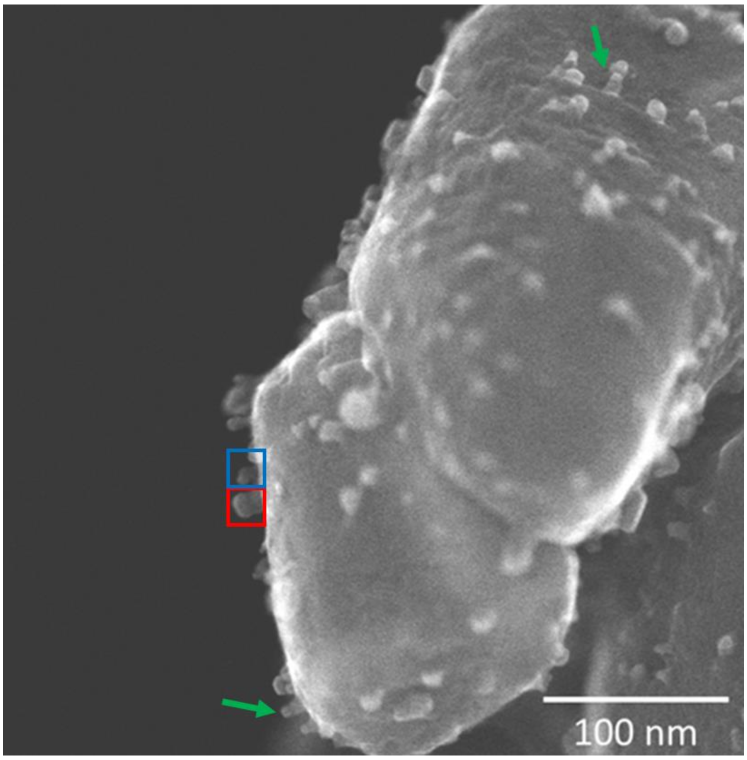

Figure S11: SE E-STEM overview image illustrating the distribution of nanorods anchored to nanoparticles. Red ROI marks the region that is displayed in **Error! Reference source not found.**Figure S11. Blue ROI highlights the region that is represented in Figure S9. Green arrows mark ZnO nanorods, which have not been imaged *in situ*.

Decomposition of ZnO – Temperature Range 500 to 800 °C

After *in situ* calcination, the high temperature behaviour of supported Pd/ZnO was studied by using *in situ* STEM during heating in vacuum. **Figure S12** shows experimental results acquired over the temperature range between 600 and 690 °C. At 600 °C, the support-particle morphology is similar to that found at 500 °C. Smaller Pd particles agglomerate to form particles with sizes of ~10 nm. The support undergoes no decomposition and has a smooth, unfaceted surface. At 660 °C, the ZnO surface starts to become faceted in the SE image (indicated by red arrows in Figure S12). At a temperature of 690 °C, an increased number of facets is observed. At the same time, the ZnO support shrinks and disintegrates, as marked by dotted blue lines. As a consequence of shrinkage of the support and an increase in temperature, the nanoparticles (green arrows in Figure S12) become more mobile and continue to agglomerate and grow in size.

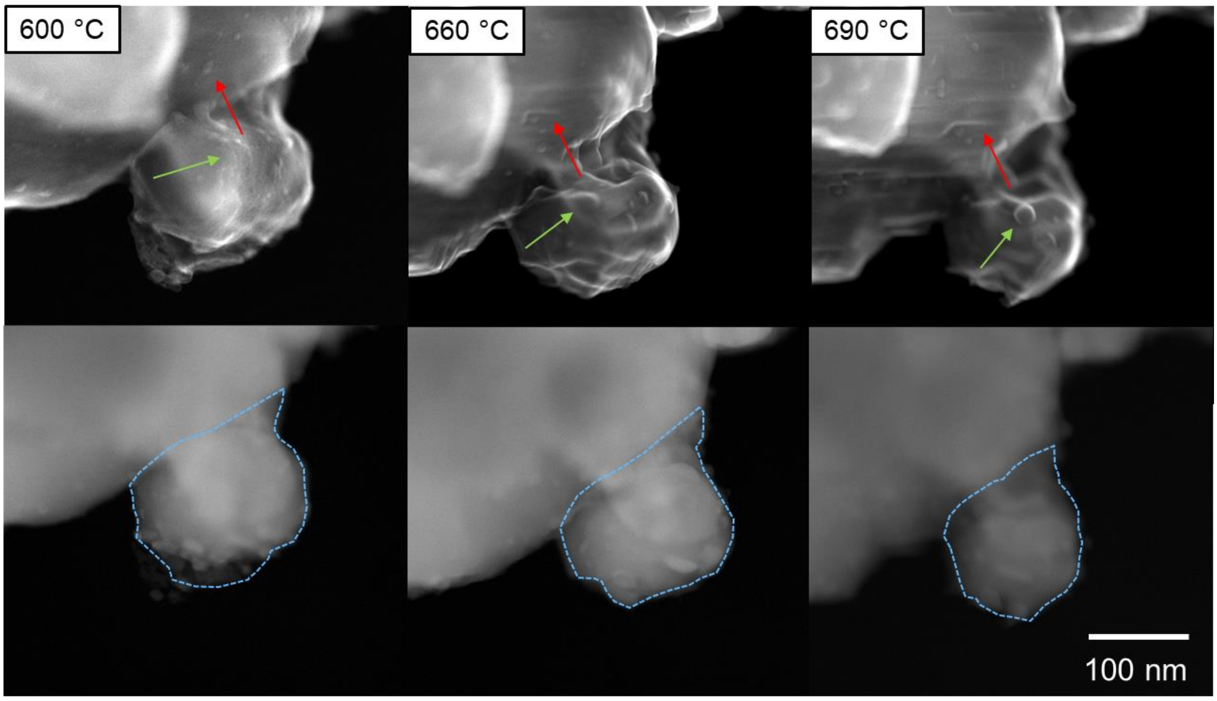

Figure S12: SE and corresponding DF *in situ* STEM image series of Pd/ZnO decomposition between 600 and 690 °C. The red arrows indicate faceting of the ZnO support; the green arrows highlight the agglomeration of Pd particles; the dotted blue lines illustrate shrinkage of the ZnO support.

The morphological changes in the Pd/ZnO system triggered by temperatures between 700 and 790 °C are summarized in **Figure S13**. An increase in temperature above 700 °C further destabilizes the system. Between 700 and 760 °C, the support decomposes until the region of interest vanishes at around 770 °C. Smaller particles, which are more exposed to the atmosphere, disappear more rapidly. Thus, the speed of decomposition of the ZnO appears to be affected by the size and exposure of the particles. At the same temperature, Pd particles grow due to decrease in support volume. Simultaneously, Pd starts to form facets at ~740 °C, as indicated on the particle marked by a red circle.

Smaller and more isolated regions are affected more strongly due to their higher surface area and surface to volume ratio. The strong decomposition of the ZnO support at temperatures of ≤ 800 °C may seem to be unanticipated, since ZnO is known to melt at ambient pressure at ~ 1975 °C.[68] However, ZnO starts to dissociate to Zn and O_2 at elevated temperature, from ~700 °C, at the surface. [69] As a consequence of the dissociation and high vapor pressure of Zn, metallic Zn melts and starts to evaporate, since its melting point of $T_{melt} = 419$ °C[70] has been surpassed, and elemental oxygen desorbs. The residual ZnO support restructures to an energetically most favourable shape and forms facets.

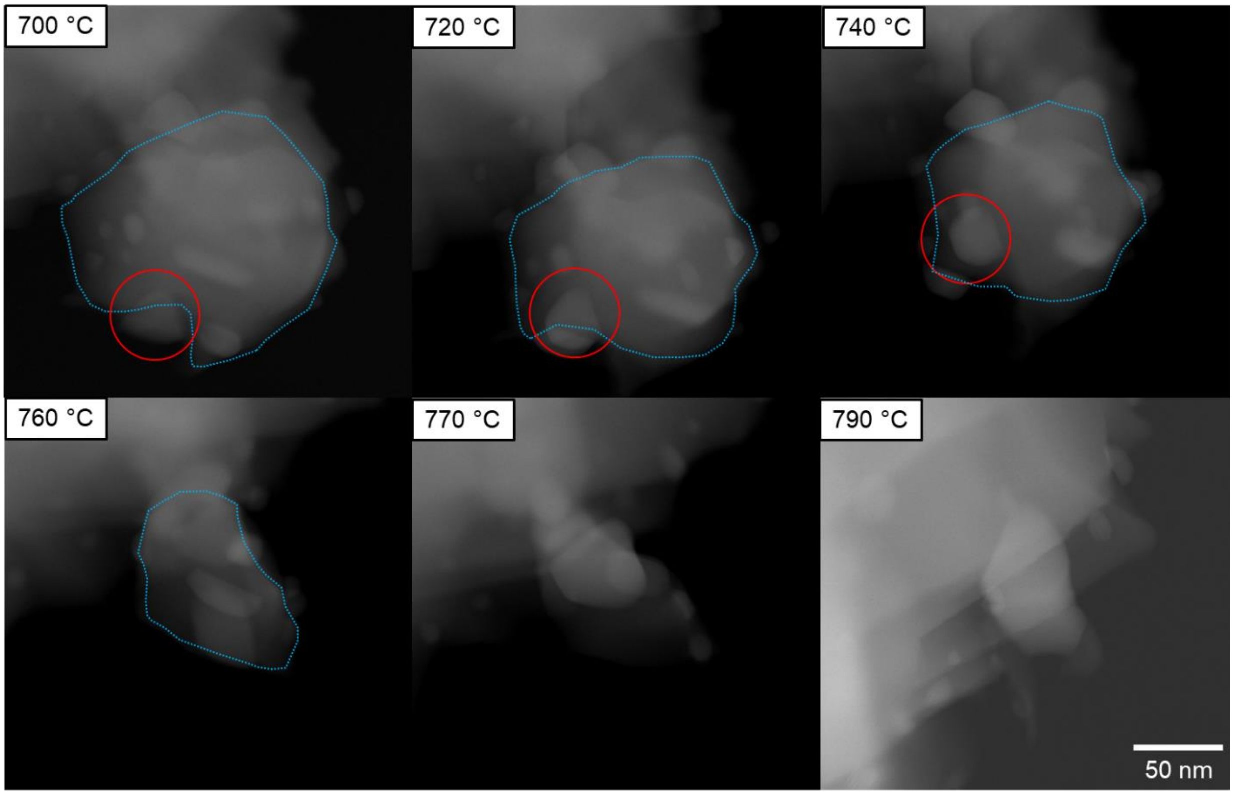

Figure S13: DF *in situ* STEM image series of the high temperature properties of Pd/ZnO between 700 and 790 °C. The dotted blue lines highlight shrinkage of the ZnO support. The red circles mark faceting of a Pd particle.

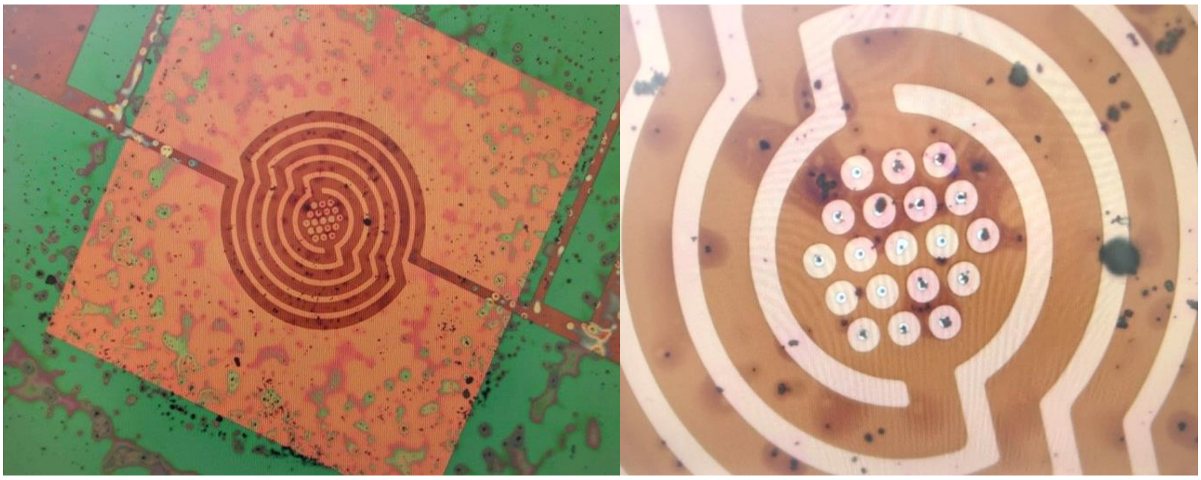

Figure S14: Optical microscopy image of a MEMS heating chip (Norcada Inc., Edmonton, Canada). Left: heating spiral with contacting, 5x magnification. Right: 50x magnification of SiN-windows in the centre of the heating spiral. The supported $Pd(NO_3)_2$/ZnO specimen is visible as dark particles positioned on the windows.

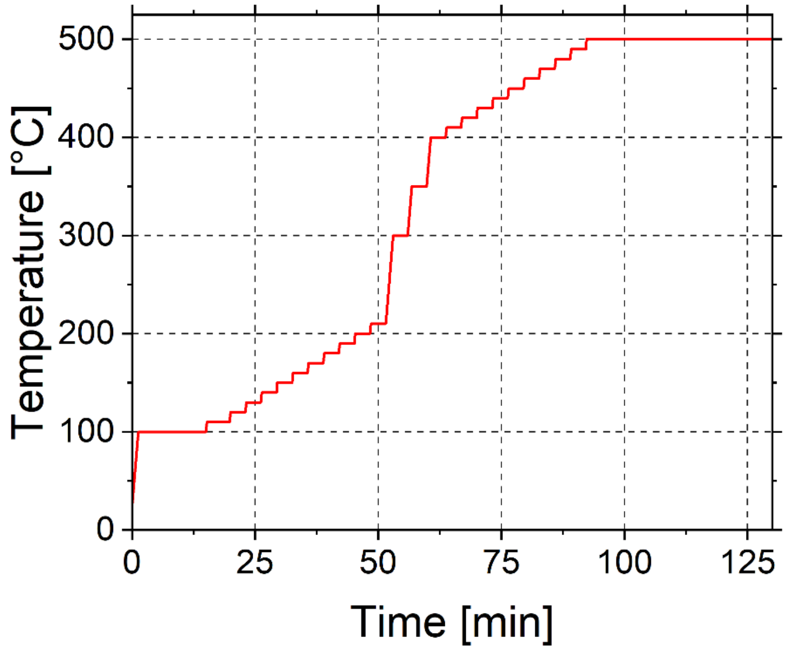

Figure S15: Heating profile of an *in situ* calcination E-STEM experiment.

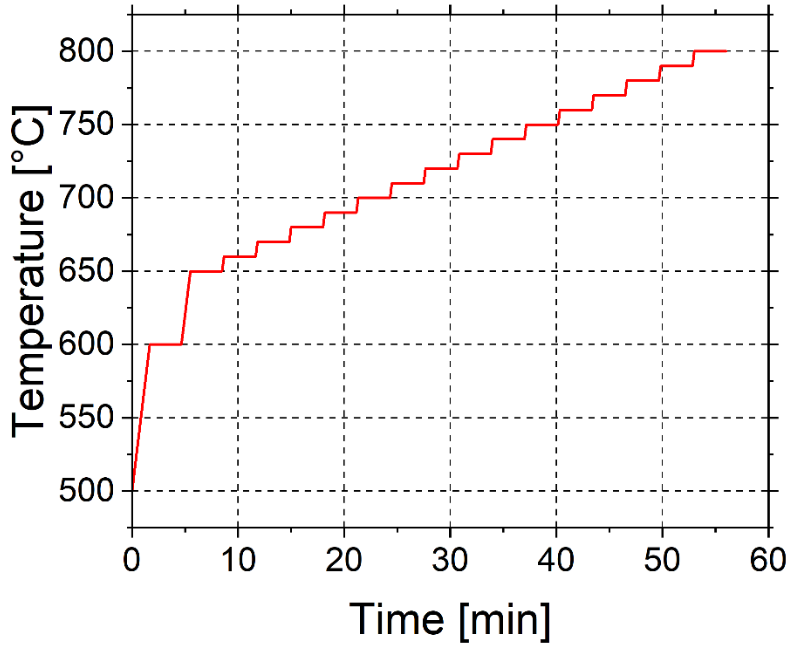

Figure S16: Illustration of a heating ramp during the present *in situ* STEM experiment between 500 and 800 °C used for imaging the high temperature behaviour of the Pd/ZnO samples.

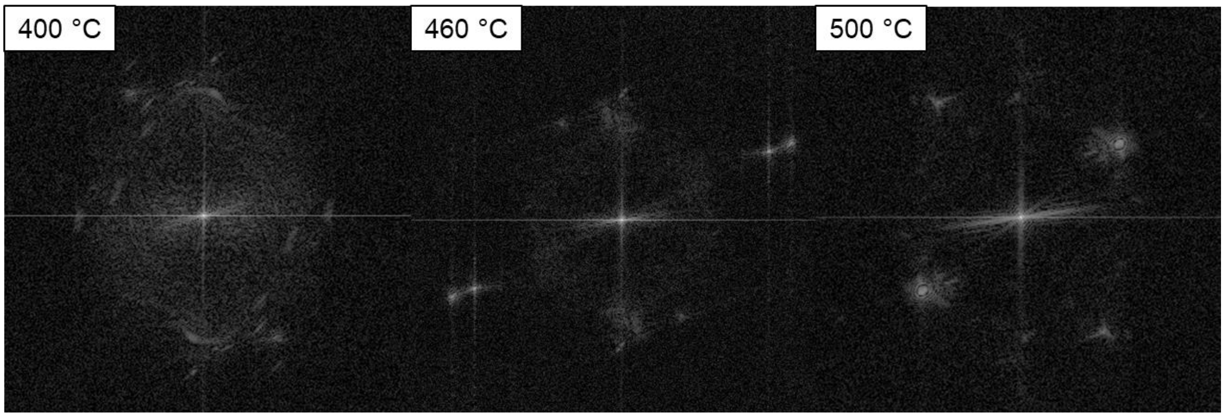

Figure S17: Series of FFTs based on images, which are shown in **Error! Reference source not found.**.

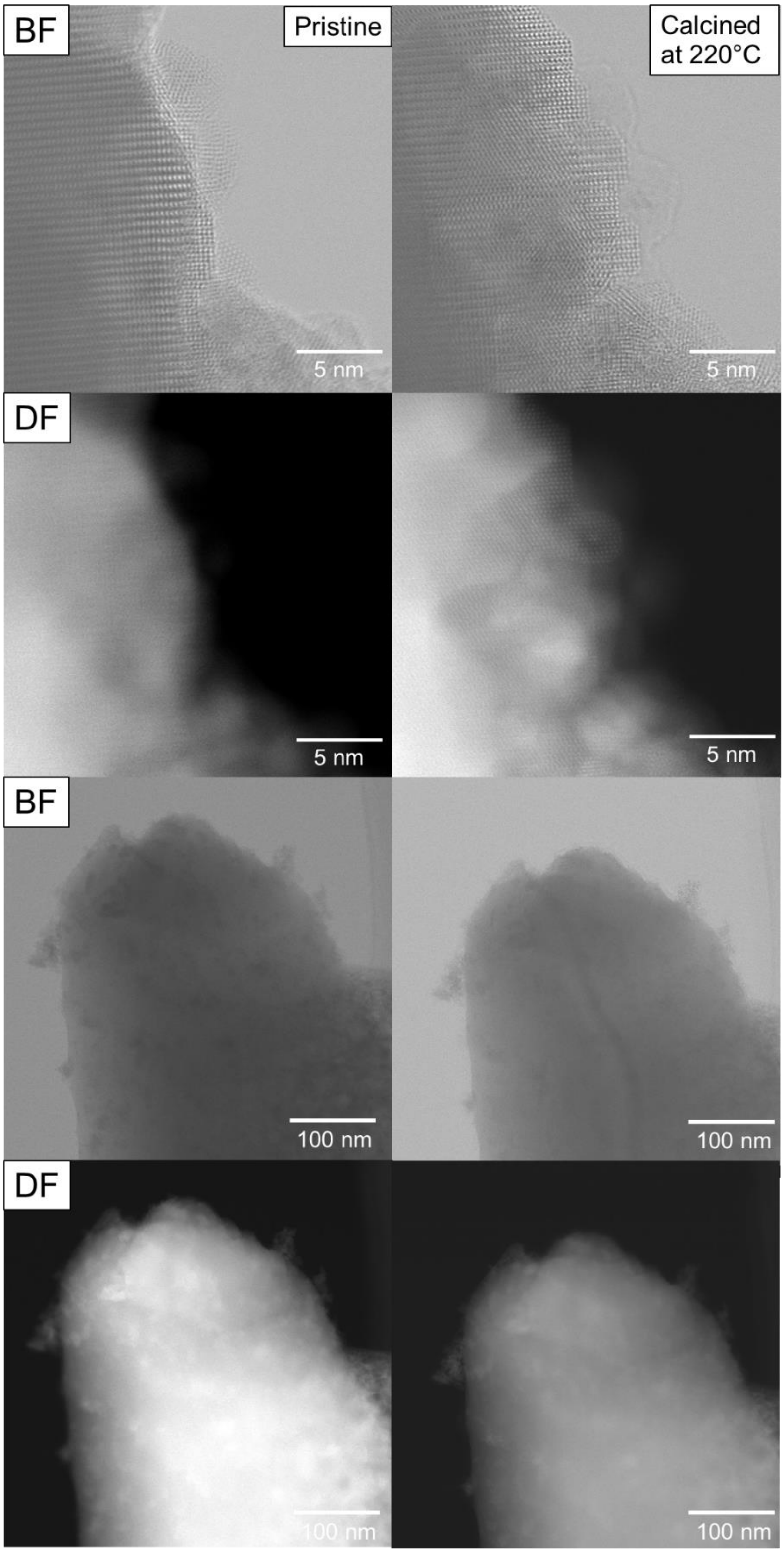

Figure S18: BF and DF images of pristine sample (left column) and the identical location after calcination at 220 °C in the E-STEM (right column). The illustrated locations were not exposed to the electron beam during the *in situ* calcination experiment and imaged only before and after the reaction in vacuum at 60 °C.

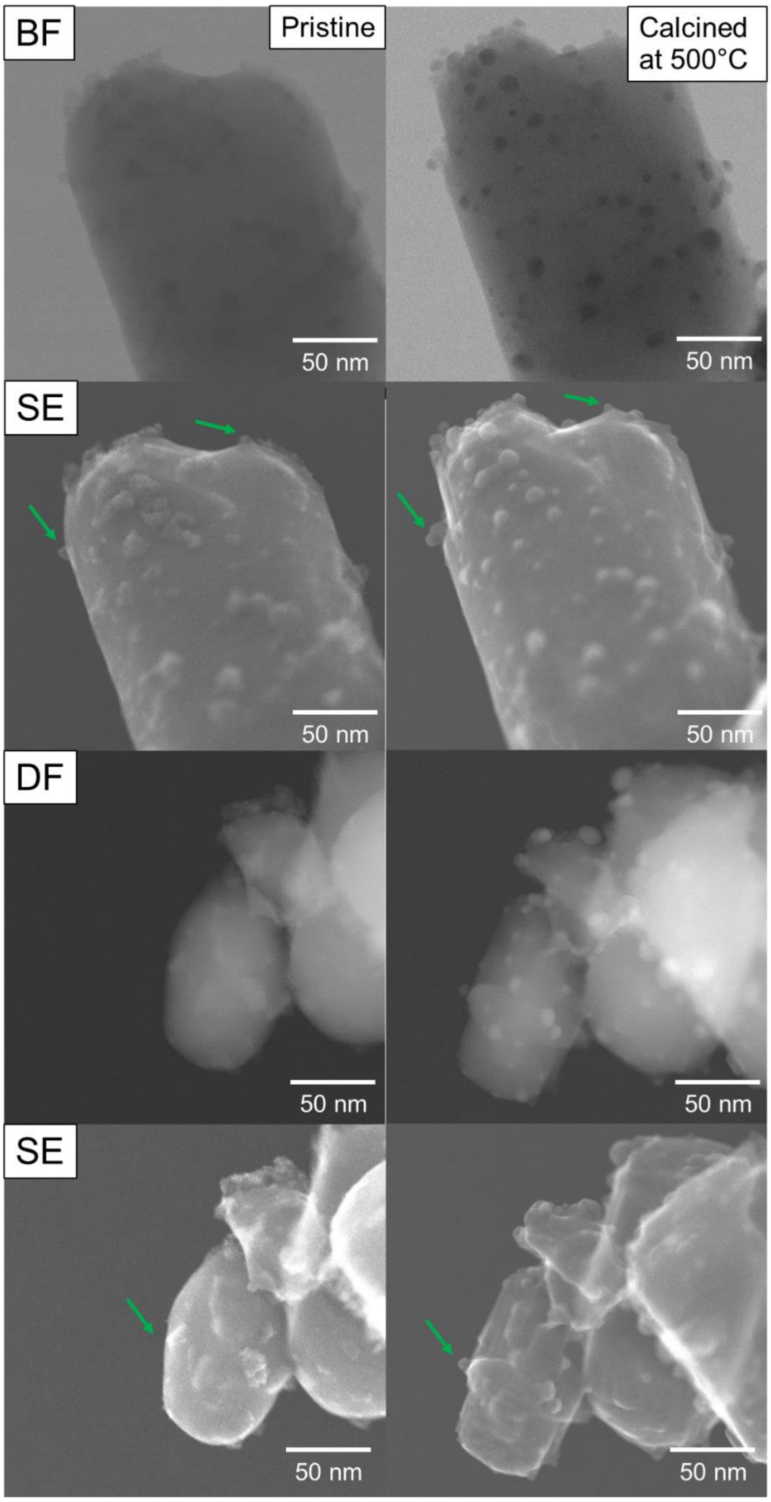

Figure S19: BF, SE and DF images of pristine sample (left column) and the identical location after calcination at 500 °C in the E-STEM (right column). The illustrated locations were not exposed to the electron beam during the in situ calcination experiment and imaged only before and after the reaction in vacuum at room temperature. The green arrows indicate locations where ZnO nanorods formed.
